# Insights from equitable governance assessments in conservation areas around the world

**DOI:** 10.1111/cobi.70101

**Published:** 2025-07-05

**Authors:** Naira Dehmel, Kate Schreckenberg, Phil Franks, Nikoleta Jones, Francesca Booker, Cosmas Lambini, Ruth Pinto, Alejandra Cely‐Gómez, Ishmael Chaukura, Donald Chilengwe Chikumbi, Phanith Chou, Ioli Christopoulou, Retche P. Colegado, Juliana Echeverri, Emelda Miyanda Hachoofwe, Kalyan Hou, Corinne Samantha Julie, Reuben Lendira, Rodgers Lubilo, Bertille Mayen, Joyce Nyaruai Mbataru, Teresa Morales, Nguyen Viet Dung, Vincent Oduka Oluoch, Jean‐Aimé Razafindra‐Paul, Natalie J. Robinson, Constance M. Schéré, Samwel Shaba, Medard Twinamatsiko

**Affiliations:** ^1^ Department of Geography King's College London London UK; ^2^ International Institute for Environment and Development London UK; ^3^ Institute for Global Sustainable Development University of Warwick Coventry UK; ^4^ GIZ Bogotá Colombia; ^5^ Community Campfire Association of Zimbabwe Harare Zimbabwe; ^6^ Department of Governance The Copperbelt University Kitwe Zambia; ^7^ Faculty of Development Studies Royal University of Phnom Penh Phnom Penh Cambodia; ^8^ The Green Tank Athens Greece; ^9^ Social Sciences Department Bukidnon State University Malaybalay Philippines; ^10^ IUCN South America Quito Ecuador; ^11^ RECOFTC Phnom Penh Cambodia; ^12^ Nature Seychelles Roche Caiman Seychelles; ^13^ Sera Wildlife Conservancy Samburu County Kenya; ^14^ Conservation Coalition Zambia Lusaka Zambia; ^15^ GIZ Yaoundé Cameroon; ^16^ Kenya Wildlife Conservancies Association Nairobi Kenya; ^17^ Conservación Amazónica – ACEAA La Paz Bolivia; ^18^ WWF Vietnam Hanoi Vietnam; ^19^ Independent Menabe Madagascar; ^20^ Gunung Palung Orangutan Conservation Program West Kalimantan Indonesia; ^21^ Department of Anthropology Rutgers University New Brunswick New Jersey USA; ^22^ Honeyguide Arusha Tanzania; ^23^ Department of Environment and Livelihood Support Systems Mbarara University of Science and Technology Mbarara Uganda

**Keywords:** area‐based conservation, justice, kunming–Montreal Global Biodiversity Framework, protected areas, social equity, áreas protegidas, conservación basada en el área, equidad social, justicia, Marco Mundial Kunming‐Montreal de la Diversidad Biológica

## Abstract

Global policy commitments to ensure that protected and conserved areas (PCAs) are equitably governed have increased interest in empirically assessing and analyzing social equity. Although numerous assessments have been conducted in individual PCAs, there is limited empirical insight into equity as a multidimensional concept beyond the site level. We investigated the distributional, procedural, and recognitional equity challenges associated with the governance of PCAs and determined whether the identified challenges differed according to governance type and actor group. We conducted a meta‐level analysis of equitable governance assessments conducted at 37 PCAs in 19 countries that form part of the new SAGE (Site‐level Assessment of Governance and Equity) database. SAGE is a participatory tool for site‐level actors to systematically discuss and assess equity dynamics at their PCAs. We found a large variation in assessment results across the sites. Mitigating the negative impacts of conservation on local communities was most often identified as the biggest challenge. In general, equity assessments tended to be slightly more positive for PCAs governed by and with Indigenous Peoples and local communities than those governed purely by government agencies. Evaluations of different actors often revealed substantial differences in opinion on specific governance issues. In particular, evaluations of PCA decision‐makers tended to be more positive than those of PCA users. As an early‐stage exploration of the growing SAGE database, our findings provide proof of concept that tools for assessing and improving PCA governance gain value from taking multidimensional approaches and need to consider different actors’ views. Although the growing SAGE database holds potential for further insights on how equity is perceived across governance types, ecosystems, and geographical regions, the primary objective of SAGE needs to remain understanding and advancing equity at the site level.

## Abstract (es)

Información de las evaluaciones de gobernanza equitativa en áreas de conservación de todo el mundo

**Resumen:**

Los compromisos políticos mundiales para garantizar que las áreas protegidas y conservadas (APC) se gobiernen de forma equitativa han aumentado el interés por evaluar y analizar empíricamente la equidad social. Aunque se han llevado a cabo numerosas evaluaciones en áreas protegidas y conservadas individuales, la comprensión empírica de la equidad como concepto multidimensional más allá del nivel del sitio es limitada. Investigamos los problemas de equidad distributiva, procedimental y de reconocimiento asociados a la gobernanza de las APC y determinamos si los problemas identificados diferían según el tipo de gobernanza y el grupo de actores. Realizamos un metaanálisis de las evaluaciones de gobernanza equitativa llevadas a cabo en 37 APC de 19 países que forman parte de la nueva base de datos SAGE (evaluación a nivel de sitio para la gobernanza y la equidad). SAGE es una herramienta participativa para que los agentes locales debatan y evalúen sistemáticamente la dinámica de la equidad en sus APC. Los resultados de las evaluaciones varían mucho de un lugar a otro. Identificamos con mayor frecuencia a la mitigación de los efectos negativos de la conservación en las comunidades locales como el mayor reto. En general, las evaluaciones de equidad tendieron a ser ligeramente más positivas para las APC gobernadas por y con pueblos indígenas y comunidades locales que para las gobernadas puramente por organismos gubernamentales. Las evaluaciones de los distintos actores revelaron a menudo diferencias sustanciales de opinión sobre cuestiones específicas de gobernanza. En particular, las evaluaciones de los responsables de la toma de decisiones de las APC tendieron a ser más positivas que las de los usuarios de las APC. Como exploración inicial de la creciente base de datos SAGE, nuestros hallazgos proporcionan una prueba de concepto de que las herramientas para evaluar y mejorar la gobernanza de las APC obtienen valor al adoptar enfoques multidimensionales y necesitan considerar los puntos de vista de los diferentes actores. Aunque la creciente base de datos SAGE tiene potencial para obtener más información sobre cómo se percibe la equidad en los distintos tipos de gobernanza, ecosistemas y regiones geográficas, el objetivo principal de SAGE debe seguir siendo comprender y promover la equidad a nivel de sitio.

## INTRODUCTION

The importance of equitably governing protected and conserved areas (PCAs) was formally recognized in global conservation policy through the Kunming–Montreal Global Biodiversity Framework (GBF), adopted by the Convention on Biological Diversity (CBD) in 2022a. The GBF's Target 3 to expand the world's conservation areas to 30% of all land, water, and sea by 2030 includes a commitment to establish “equitably governed systems,” “ensuring…sustainable use” and “recognizing and respecting the rights of Indigenous Peoples and local communities, including over their traditional territories” (CBD, 2022a, p. 9). The activism, research, and political engagement of Indigenous Peoples, researchers, civil society, and human rights organizations has played a pivotal role in contesting the common injustices in dominant approaches to area‐based conservation (e.g., Agrawal et al., 2021; Armitage et al., 2020; Brockington, 2002; Corson et al., 2020; Human Rights in Biodiversity Working Group, 2022; International Indigenous Forum on Biodiversity, 2022; Minority Rights Group International et al., 2020; Survival International, 2024). With a history of imposed PCA designations, area‐based conservation has often displaced and evicted Indigenous Peoples and local communities (IP&LC) (e.g., Brockington & Igoe, 2006; Schmidt‐Soltau & Brockington, 2007). Additionally, its management practices, including militarized enforcement and reliance on Western scientific ontologies, have been criticized for perpetuating colonial and racist legacies (e.g., Adams, 2019; Brockington, 2002; Corson & Campbell, 2023). Beyond the moral implications of such social justice concerns, increasing evidence supports the instrumental argument for equitably governing natural resources as a means of ensuring management effectiveness and ecological outcomes of conservation measures (Dawson et al., 2021; Dawson, Coolsaet, Bhardwaj, Booker, et al., 2024; Dawson, Coolsaet, Bhardwaj, Brown, et al., 2024; Hampton‐Smith et al., 2024; Oldekop et al., 2016; Pinto & Dawson, 2023).

Equity in the context of conservation is commonly characterized as having 3 dimensions: distribution, procedure, and recognition (CBD, 2018; Franks et al., 2018; Friedman et al., 2018; Hampton‐Smith et al., 2024; Loos et al., 2023; Pickering et al., 2022; Zafra‐Calvo et al., 2017). Rooted in social and environmental justice scholarship (e.g., Fraser, 2008; Schlosberg, 2007), these 3 dimensions recognize that social justice concerns do not relate only to how fairly conservation benefits and burdens are distributed. Rather, they are intrinsically linked to and experienced through the processes by which decisions are made, implemented, and contested, and the degree to which actors’ rights, identities, knowledges, values, and institutions are recognized and respected (Loos et al., 2023; Martin et al., 2016). Nonetheless, most empirical academic research on social dimensions of conservation has traditionally focused on the distributional dimension (Friedman et al., 2018), with only recent attention to procedure (Hampton‐Smith et al., 2024).

Formulating normative principles underlying the 3 dimensions has allowed for social equity to be operationalized as a multidimensional concept (Franks et al., 2018; Schreckenberg et al., 2016; Zafra‐Calvo et al., 2017). Academic and applied assessments of equity and governance are increasingly using such a multidimensional and principle‐based approach (e.g., Bennett et al., 2020; Echeverri et al., 2021; Schéré et al., 2021; Zafra‐Calvo et al., 2019; Zhang et al., 2024, 2025). Bennett et al. (2020), for example, conducted an equity survey based on 20 principles with small‐scale fishers in marine protected areas in the Mediterranean Sea and through this were able to show strengths and weaknesses across the dimensions and sites. Nonetheless, such multidimensional empirical assessments of social equity have primarily been conducted at individual PCAs or as by Bennett et al. (2020) across PCAs in a specific region. Besides systematic literature reviews (Friedman et al., 2018; Hampton‐Smith et al., 2024), only Zafra‐Calvo et al. (2019) have conducted a global‐level analysis of data collected through an online questionnaire distributed among mainly PCA and government staff, nongovernmental organization (NGO) practitioners, and academics. Overall, there is still very limited empirical insight into the state and dynamics of multidimensional equity across sites and regions.

The International Union for Conservation of Nature (IUCN) distinguishes among 4 PCA governance types: governance by government, shared governance, private governance, and governance by IP&LC (Borrini‐Feyerabend et al., 2013). A global‐level review of how PCAs under different governance types affected the well‐being of local people showed that most reported costs come from PCAs governed by governments, whereas most socioeconomic benefits are reported for PCAs under shared governance, followed by those under governance by IP&LC (Oldekop et al., 2016). Similarly, several literature reviews by Neil Dawson and colleagues show more positive social (and ecological) outcomes reported by conservation efforts where IP&LC have substantial influence over decision‐making and more negative social outcomes in initiatives controlled by external organizations (Dawson et al., 2021; Dawson, Coolsaet, Bhardwaj, Booker, et al., 2024; Dawson, Coolsaet, Bhardwaj, Brown, et al., 2024; Pinto & Dawson, 2023). At the same time, however, conservation measures that recognize IP&LC rights or devolve decision‐making, such as shared governance arrangements, community‐based conservation, and conservation projects in Indigenous territories, in many places have also been prone to elite capture, imposition of top‐down practices, and dominant Western ontologies (Armitage et al., 2020; Corson et al., 2020; Eichler & Baumeister, 2022; Igoe & Croucher, 2007; Kashwan, 2013). Even though certain principles of procedural equity, such as participation of local populations in decision‐making, are in theory essential elements of shared and IP&LC‐governed PCAs, governance type does not necessarily translate into governance quality and equitability in practice (Franks et al., 2018). In addition to the evidence on diverging social and ecological outcomes by governance type, insights are missing on social equity from a multidimensional perspective in different governance types.

In contrast to other evaluation criteria, such as project outputs, effectiveness, transparency, and equality, social equity is understood to be inherently subjective (Gurney et al., 2021; Schreckenberg et al., 2016; Sikor et al., 2014). Social equity in the sense of fairness implies an emotional response by individuals to their experience of a situation (Hamann et al., 2018). Whether or not a project and its outcomes are perceived to be equitable often cannot be rationally or objectively understood yet can still be important for an individual's support or rejection of it (Bennett et al., 2019, 2020; Hamann et al., 2018; Kuhn, 2011; Sikor et al., 2014). Although normative principles of what conceptually constitutes equity may be established theoretically, which element is more or less important is based on cultural and individual value systems, and the evaluation of the same element can be different depending on individuals’ positionalities and perspectives (Franks, 2021; Gurney et al., 2021; Loos et al., 2023). In recognition of the inherent subjectivity of equity, recent equity assessments engage with different stakeholders’ and rightsholders’ perspectives (Echeverri et al., 2021; Schéré et al., 2021; Zafra‐Calvo et al., 2019) or focus exclusively on perceptions of local people (e.g., Bennett et al., 2020; Zhang et al., 2024, 2025). A site‐level assessment across 3 marine protected areas in the Irish Sea, for example, showed a substantial disconnect between the views of people involved in PCA management and other stakeholders (Schéré et al., 2021). Similarly, Zafra‐Calvo et al. (2019) found some significant differences in the evaluations between different respondents to their global survey on free prior and informed consent and access to justice. However, their dataset included only a very limited number of representatives from local populations affected by PCAs themselves. Few other data explore differences in opinions between actor groups.

Despite the rapidly growing interest in assessing social equity in PCAs, both for site‐level evaluation and learning but also upward reporting against new global targets (Bennett, 2016; Moreaux et al., 2018; Zafra‐Calvo et al., 2017), there is still limited empirical insight into different experiences of multidimensional equity at PCAs of different governance types across sites and regions. We addressed this gap by conducting the first overarching analysis of a novel emerging dataset of equitable governance assessments in which the SAGE (Site‐level Assessment of Governance and Equity) tool was used. SAGE is one of the suggested indicators for GBF Target 3 (CBD, 2022b) and is a tool for site‐level stakeholders and rightsholders to self‐assess equitable governance in conservation areas of any governance type and to identify, plan, and implement actions for improvement (IIED, 2023). It allows different site‐level actors to discuss and document their diverging perceptions and opinions of a set of principles covering multiple dimensions of social equity. Although primarily intended for use at the site level, the standardized methodology of SAGE allows for the resulting data to be analyzed at a meta‐level and lessons to be drawn about equitable governance of PCAs more widely.

As a contribution to the policy‐oriented literature on equitable governance in conservation, we analyzed the data from 37 SAGE assessments across 19 countries. To address the outlined empirical gaps, we asked the following questions: across the various principles of social equity, what are the most common equity challenges reported in the PCAs; are there any differences in SAGE assessments between PCAs under shared and IP&LC governance compared with those governed by governments; and how is equitable governance perceived by different actors?

Given that SAGE simplifies equity challenges across a diverse range of contexts and people may be culturally inclined to respond more or less positively, SAGE scores at different sites are not necessarily comparable in absolute terms. Rather than normatively comparing how well one PCA is doing relative to another, we aimed to identify patterns across sites. SAGE assessments are conducted only in PCAs where there are no overt conflicts, the risk is low for SAGE to create or exacerbate conflict, all key actors are willing to engage fully in the assessment, and PCA managers and at least some other actors are willing to address equity challenges (IIED, 2023, p. 11). This means that our findings are not representative of the full spectrum of PCA contexts; rather, they relate to conservation areas that are already comparatively conducive to addressing IP&LC needs and priorities. Nonetheless, even within these sites, equity challenges persist.

## METHODS

### SAGE

SAGE is a tool used to evaluate equitable governance in PCAs (IIED, 2023). It is based on a multiple‐choice questionnaire, responses to which can be aggregated into equity scores. Its process allows for a facilitated interactive discussion between the various relevant actors at a site. Following preparations led by a facilitator, including a stakeholder analysis (described in the SAGE manual [IIED, 2023, p. 12]), adaptation of the questionnaire to the local context, and seeking actors’ consent, the actors themselves conduct the assessment. First, different actor groups, identified as sharing similar interests and influence, convene separately to respond to the questionnaire and discuss their experiences and opinions in relation to each question. This is followed by a synthesis workshop where the different groups come together to exchange and discuss their results.

SAGE is based on 10 principles (Table 1). The first 8 underly the equity dimensions of recognition, procedure, and distribution, and the last 2 relate to other aspects of governance quality, namely, effectiveness of collaboration and achievement of objectives. They are based on previous formulations of equity principles, especially by Schreckenberg et al. (2016), Franks et al. (2018), and the IUCN principles of good governance for protected areas (Borrini‐Feyerabend et al., 2013). The SAGE questionnaire includes 5 multiple‐choice questions for each principle, one for the facilitator to respond to and 4 for the participating actor groups to discuss and score. The questions for facilitators assess to what degree definitions, documentation, and studies are in place to support each principle. The questions for the site‐level actors, in contrast, evaluate their perception of and experience with specific governance issues under each principle. There are 4 possible responses per question that are normatively ordered from least to most equitable and can be translated into equity scores on a 0–3 scale (see manual for SAGE questions [IIED, 2023]). For identified weaknesses, or significant disparities in actors’ opinions, the participants brainstorm potential actions for improvement. The SAGE results consist of quantitative equity scores by each actor group that can be aggregated for each principle. In addition, qualitative notes are taken on the justifications for each score, as well as ideas for action. Although typically convened by one of the site‐level actors, such as PCA managers, NGOs, funders, or academics, SAGE assessments are facilitated by an experienced, usually external, facilitator seen as neutral by site‐level actors and who has the necessary skills for navigating power imbalances between actors.

**TABLE 1:** Equity principles in SAGE (Site‐level Assessment of Governance and Equity).

Equity dimension	Equity principle
Recognition	P01 – Respect for resource rights and human rights of community members
	P02 – Respect of all relevant actors and their knowledge, values, and institutions
Procedure	P03 – Effective participation of all relevant actors in decision‐making
	P04 – Transparency, information sharing, and accountability for actions and inactions
	P05 – Access to justice including effective dispute resolution processes
	P06 – Fair and effective law enforcement
Distribution	P07 – Effective mitigation of negative impacts on community members
	P08 – quitable sharing of benefits among relevant actors
Other	P09 – Achievement of conservation and other objectives
	P10 – Effective coordination and collaboration among actors, sectors, and levels

*Source*: IIED (2023, p. 17).

### Data preparation

**FIGURE 1:**
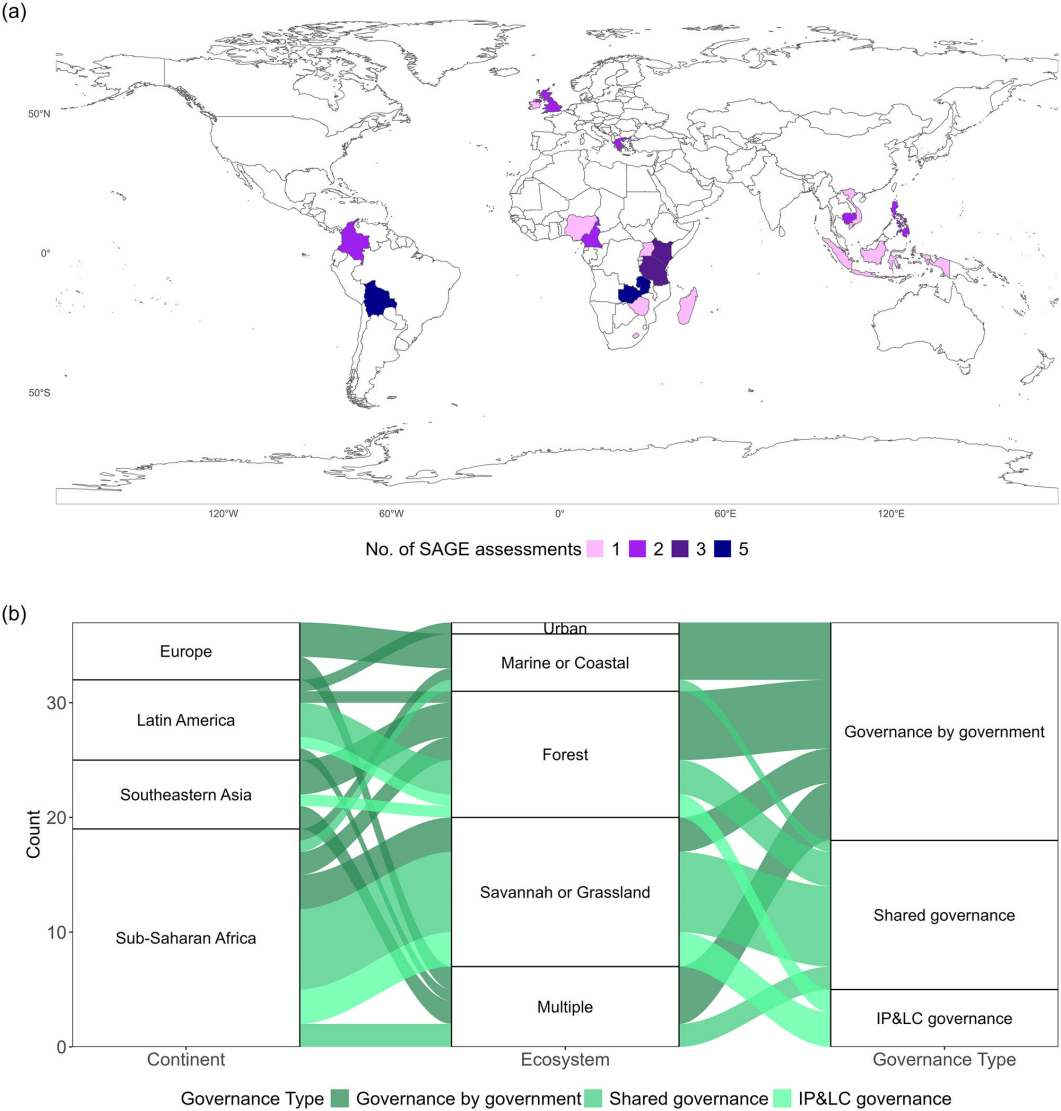
(a) Countries where the included SAGE (Site‐level Assessment of Governance and Equity) assessments were conducted and (b) summary of key site characteristics (IP&LC, Indigenous PPeoples and local communities).

Our analyses included all the SAGE assessments conducted from November 2019 to March 2023 for which the convenors or facilitators shared their data with us for use in this study (37 of 54 sites completed by then). The included sites span different continents, ecosystems, and governance types (Figure 1). The remaining sites were not included either because convenors or facilitators did not respond to the permission request (*n* = 10), gave permission but did not share the data (*n* = 4), or the data were deemed too sensitive to be shared (*n* = 3). This self‐selection was the most ethical and feasible option but implies the possibility of bias toward sites where the process and results were less contentious.

### SAGE principles

**FIGURE 2:**
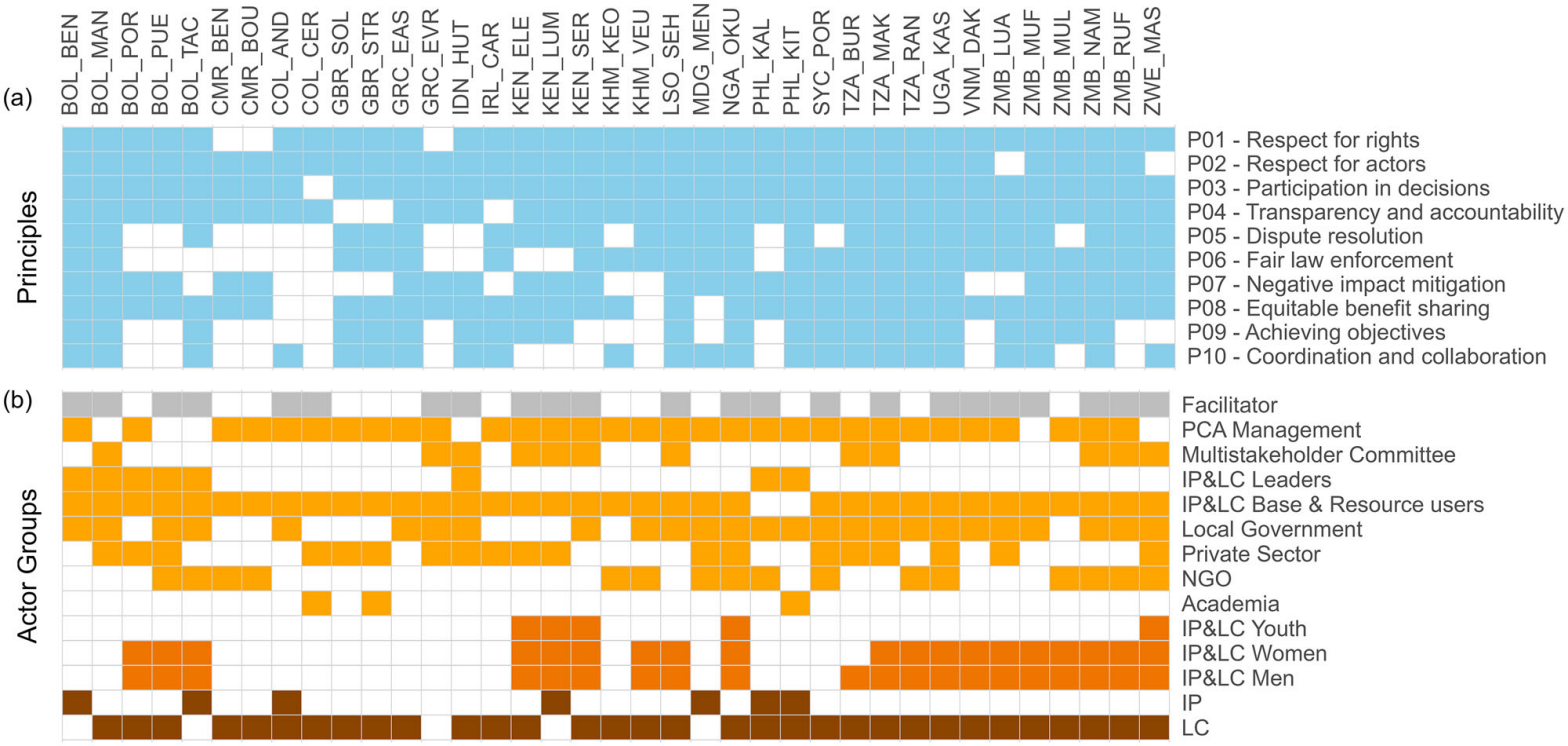
(a) Included principles and (b) actor groups at each SAGE (Site‐level Assessment of Governance and Equity) site (dark orange, subcommunity actor groups grouped above within s the Indigenous Peoples and local communities [IP&LC] base and resource users group; brown, Indigenous Peoples [IP] and local communities [LC] grouped above within IP&LC leaders and IP&LC base and resource user groups; codes at top, site country codes and the first 3 letters of the site name; PCA, protected and conserved area; NGO, nongovernmental organization).

A SAGE assessment normally includes 8 of the 10 principles (IIED, 2023). Yet, depending on time and budget, convenors and facilitators often decide to include fewer principles or indeed all 10 (Figure 2a). SAGE facilitators may adapt specific questions so that they make sense within their site's context. To ensure such adaptations both adhere closely to the principle and reflect its breadth sufficiently, the SAGE manual provides themes that any new questions should relate to (Table 2). However, some SAGE facilitators adapted the questionnaire to a greater degree or excluded questions altogether. In addition, 10 of the 37 sites used an earlier version of the SAGE questionnaire that was built on the same equity framework and principles but did not include the same number of questions per principle nor the additional questions for facilitators to respond to. To ensure the included SAGE assessments were sufficiently similar to be analyzed together, we compared all available questionnaires and conducted all quantitative analyses at the principle level (rather than by question), excluded data from any questions with a different meaning to the given theme, and excluded data for which there were <2 questions or data points for a principle (details in Appendix S1).

**TABLE 2:** Example questions and themes under equity principle on mitigation of negative impacts (P07).

	Theme	SAGE question
Question for facilitator	Assessment of negative social impacts	7.1 Have there been any recent studies of negative impacts of the PCA and its conservation on community members?
Questions for actor groups	Implementation of mitigation actions – first example	7.2 Do organizations responsible for reducing impacts on communities of [insert negative impact] act as they are supposed to?
	Effectiveness of mitigation actions – first example	7.3 How successful are their actions in reducing the impacts on communities of [insert negative impact]?
	Implementation of mitigation actions – second example	7.4 Do organizations responsible for reducing impacts on communities of [insert negative impact] act as they are supposed to?
	Effectiveness of mitigation actions – second example	7.5 How successful are their actions in reducing the impacts on communities of [insert negative impact]?

*Source*: IIED (2023, p. 51).

### Governance type

The dataset includes PCAs in 3 of the 4 IUCN governance types: governance by government, shared governance, and IP&LC governance (Figure 1b). The governance type for each site was derived from, in order of priority depending on availability, SAGE site profiles and reports, the World Database of Protected Areas, IIED staff, facilitators or convenors, or an online search.

### Actor groups

During the SAGE preparation phase, following a stakeholder analysis, actors identified as having significant interest or influence in the PCA are grouped into 3–7 actor groups. The number of actor groups per site and how homogenous they are depended on the complexity of actors and budget and time constraints.

Across all the included sites, actor groups and their names varied widely. To reduce the list of actor groups for this analysis, we clustered them into 8 categories (Figure 2b). Where the provided names for actor groups were not self‐explanatory, we reviewed SAGE reports, consulted IIED staff and facilitators, or conducted an online search.

There were several groups that included multiple actors, mostly combining PCA management, government, NGOs, and academics. Rather than excluding such overlapping groups, we assigned the category that was represented most prominently within the group (details in Appendix S2).

Most SAGE assessments distinguished between different types of local community members, including mainly distinctions between gender, age, Indigenous versus non‐Indigenous, or different forms of resource use (e.g., hunters, fishers or, especially in the European context, recreational visitors). However, for most of our analyses, community opinions at a site were grouped together. We separately compared the equity scores between subcommunity groups as distinguished by gender, age, and Indigenous People (IP) versus local communities (LC). However, this was limited due to small sample sizes within the youth and IP groups (Figure 2b).

### Analyses

We computed equity scores for each principle by averaging the responses across the respective underlying questions for each actor group. We analyzed scores from facilitators and site‐level actors separately as they are not directly comparable. Statistical tests of differences between principles, actor groups, and governance types were conducted using beta‐regression models to reflect the bounded nature of scores from 0 to 3 (Ferrari & Cribari‐Neto, 2004). For this, we normalized the equity scores to fall between 0 and 1. We included random effects for country, sites, and where applicable principles to account for the nested structure of the data (Gurka et al., 2012) and applied the Bonferroni correction method to adjust the family‐wise error rate in pairwise comparisons (Lee & Lee, 2018). We conducted the analyses in R 4.2.2 (R Core Team, 2022), applying the packages glmmTMB, emmeans, ggplot2, rnaturalearthdata, and ggalluvial, and consulted ChatGPT for developing the code (OpenAI, 2023).

We qualitatively analyzed the available SAGE reports (*n* = 29) for further insights on questions that emerged through the quantitative findings using NVivo Version 14 (QSR International, 2023). We drew on the SAGE reports rather than raw qualitative data as they summarize the most important discussion points and are curated for an external audience by the SAGE facilitators (coauthors of this article). The raw qualitative data risk including sensitive issues and are not always comprehensible without contextual knowledge. The study received ethical approval from the Research Ethics Office of King's College London (LRS/DP‐20/21‐23077).

## RESULTS

### Equity assessments by principle

**FIGURE 3:**
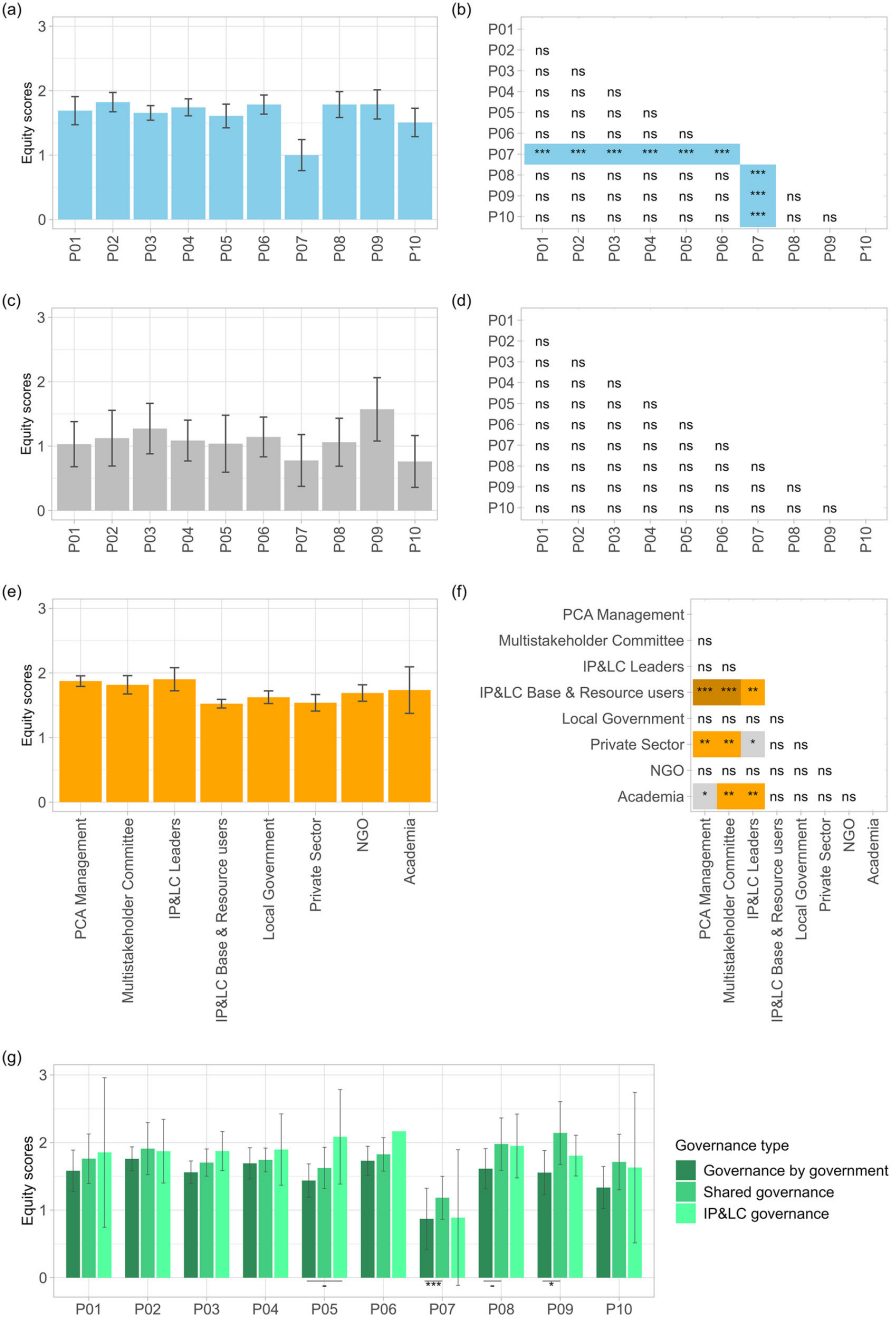
Mean equity scores (a) of actor groups by SAGE (Site‐level Assessment of Governance and Equity) principle, (c) of facilitators by principle, (e) by actor groups across all principles and (g) of actor groups by principle and governance type. In (b), (d), (f) and (g), *p* values are for pairwise comparisons between each principle, actor group or governance type, respectively (error bars, 95% confidence intervals; ns, *p* < 0.1; *, *p* < 0.5; **, *p* < 0.01; ***, *p* < 0.001; PCA, protected and conserved area; IP&LC, Indigenous Peoples and local communities; NGO, nongovernmental organization).

Overall, there was a large variation in equity assessments at each site (Appendix S3). However, across the sites, the equity scores averaged out in the middle range for most principles (Figure 3a). Notably, mitigation of negative impacts (P07) received significantly lower scores on average compared with all other principles (Figure 3b; Appendix S4), a pattern that was consistent across the different continents (Appendix S5). It received the lowest average score compared with all other principles in almost two thirds of the sites that included this principle in the assessment (17 of 27 [63%]), the second lowest in 6 sites (22.2%), and middle range in another 3 (11.1%). It was rated highest in only one Zambian site, where it was nonetheless mentioned as a main concern in the SAGE report. Accordingly, mitigation of negative impacts of the PCA on local communities was most often mentioned as a primary challenge by the SAGE reports (17 of 29 [58.6%]; Appendix S6). The reported negative impacts related primarily to human–wildlife conflict in the Kenyan, Tanzanian, and Zambian PCAs, negative repercussions of uncontrolled external extractive activities in the Bolivian PCAs, and restricted resource use without effective provisions for alternatives in Greek, Malagasy, and Indonesian PCAs (Appendix S7). In relation to human–wildlife conflict, the SAGE reports from Kenyan, Tanzanian, and Zambian wildlife conservancies, management areas, and game management reserves primarily discussed ineffective, unresponsive, delayed, and insufficient compensation measures for wildlife damage to crops, livestock, and houses. The reports attributed these problems mainly to insufficient resourcing, skills and strategic planning, lack of priority and political will, and poor community awareness of reporting mechanisms. Four Bolivian PCAs reported major challenges not with direct impacts of conservation on local communities, but rather with the ineffectiveness (or unwillingness) of the conservation actors to halt the detrimental impacts of external extractive activities, such as illegal gold mining, logging, deforestation, and forest fires. Related to access and use restrictions, reports from both a Greek and Malagasy PCA stated that there were no clear mitigation strategies in place, and any limited attempts reached only some of the affected. At the Indonesian site, existing mitigation efforts were reported to be limited du lack of resources and staffing and a lack of transparency and community participation.

After P07, the SAGE reports most often mentioned participation in decisions (P03) (12 of 29 [41.4%]) and respect for rights (P01) (10 of 29 [34.5%]) (Appendix S6) as primary challenges. Although lack of youth and women's participation was a recurring issue for P03, and P01 primarily concerned rights to resource access and use, land rights, and basic human rights, discussions of both these principles focused on the underlying challenges of limited awareness and information sharing (Appendix S7). Beyond the need for awareness raising and capacity building, several reports called for the translation of documents and spoken word into local and Indigenous languages. The fact that responses by SAGE facilitators to their questions averaged around the lower third of scores for all principles suggests that formal definitions, documentation, and studies to support equitable processes in practice were relatively poor across the sites (Figure 3c,d).

### Equity assessments by governance type

Across all principles, average equity scores were slightly higher in IP&LC and shared governance arrangements than in PCAs governed by governments alone (Figure 3g). The results were significantly different for access to justice (P05) between IP&LC and government‐governed PCAs and for mitigation of negative impacts (P07), equitable benefit sharing (P08), and achieving objectives (P09) between shared and government‐governed PCAs (Appendix S8).

### Equity assessments by actor groups

Comparing the equity scores by different actor groups showed that PCA decision‐makers tended to provide more positive evaluations than PCA users. Across all the sites and principles, PCA management, multistakeholder committees, and IP&LC leaders gave significantly higher scores than IP&LC base and resource users, the private sector, as well as academics (Figure 3e,f; Appendix S9). Although the patterns of scores between actor groups were slightly different when disaggregated by continent (with global patterns most closely reflecting the Sub‐Saharan African and Southeast Asian subsets [Appendix S10]), the sample sizes were too small to infer any interpretable differences between continents. The overall message that more powerful respondents tended to provide more positive evaluations held regardless.

Disaggregating by principle, IP&LC base and resource users and NGOs were more likely to give lower scores for participation in decisions (P03) than PCA management, multistakeholder committees, local government, and the private sector. For dispute resolution (P05), academic actors had significantly more negative evaluations than all other actor groups, whereas IP&LC base and resource users scored significantly lower than multistakeholder committees and local government. For mitigation of negative impacts (P07), IP&LC base and resource users gave significantly more negative evaluations than PCA management, IP&LC leaders, and NGOs, and the private sector scored more negatively than NGOs. For equitable benefit sharing (P08), IP&LC leaders gave significantly more positive scores than IP&LC base and resource users, local government, and NGOs. For achieving objectives (P09), NGOs gave more positive scores than the private sector. For coordination and collaboration (P10), the private sector scored significantly more negatively than PCA management, IP&LC leaders, and IP&LC base and resource users (Appendix S11). The SAGE reports provided qualitative insights into reasons for differences in opinion (Table 3).

**TABLE 3:** Explanations for differences in opinions between actor groups as described in SAGE (Site‐level Assessment of Governance and Equity) reports.

Principle^a^	Reason for differences in opinion
P03 – Participation in decisions	Some actors feel only invited when their participation is of interest to protected and conserved area (PCA) staff (Indonesian, Nigerian, and Zambian sites, *n* = 3). Lack of women and youth participation was noted only by women and youth groups themselves (Kenyan and Zambian sites, *n* = 2). Differences in scores where nongovernmental organizations (NGOs) and private sector feel fully involved, yet community members feel excluded, believe they are not valued as an important partner, and therefore perceive themselves to have little influence (Nigerian and Zambian sites, *n* = 2). Authorities gave higher scores because by law they are required to lead decision‐making (Greek site).
P05 – Dispute resolution	PCA staff, NGOs, and private sector scored highly because they just apply the law. Yet, these procedures are not perceived as meaningful processes by community members (Nigerian site). Private sector and community members scored low because of observed corruption and inappropriate conduct during law enforcement (Zambian site).
P07 – Negative impact mitigation	Conservationists value wildlife more than humans and their property (Kenyan site). Agencies do not value destruction of crops and housing for compensation even though these are considerable costs for communities (Kenyan site). Although PCA staff, NGOs, and private sector think that communities are not affected or little affected by the PCA, community members object to the creation of the PCA for its negative impacts and request zoning for continued access to land and crops (Nigerian site). Multistakeholder committee feels that things are fine, but community members and private sector say there is no action plan to address human–wildlife conflicts (Tanzanian site). Community members are not aware of compensation processes (Kenyan site).
P08 – Equitable benefit sharing	Community groups reported elite capture by community leaders of power and with access to information and benefits (e.g., employment) (Bolivian and Kenyan sites, *n* = 2). Authorities believe benefit sharing occurs according to regulations and equitably, yet other groups did not feel included in decision‐making on regulations or say authorities left little room to benefit them (Indonesian and Kenyan sites, *n* = 2)
P09 – Achieving objectives	There is a lack of awareness among community groups and private sector as opposed to PCA managers, multistakeholder committees, and NGOs regarding the objectives and the available information to gauge whether objectives are being met (Nigerian and Zambian sites, *n* = 2).
P10 – Coordination and collaboration	Private sector actors feel excluded and observe lack of information sharing (Tanzanian sites, *n* = 2).

^a^ Principles for which the quantitative analyses identified statistically significant differences between actor groups’ equity scores.

At the subcommunity level, there were some significant differences in evaluations between community youth and community men and women and between IP and LC representatives; however, there were no clear patterns. Community youth gave significantly lower scores for participation in decisions (P03) than men and for equitable benefit sharing (P08) than men and women. Yet, they gave significantly higher scores for respect for rights (P01) than men and dispute resolution (P05) than men and women (Appendix S12). Similarly, IP representatives scored significantly more negatively than LC representatives for respect for rights (P01) and coordination and collaboration (P10), and significantly more positively for participation in decisions (P03) and equitable benefit sharing (P08) (Appendix S13).

**FIGURE 4:**
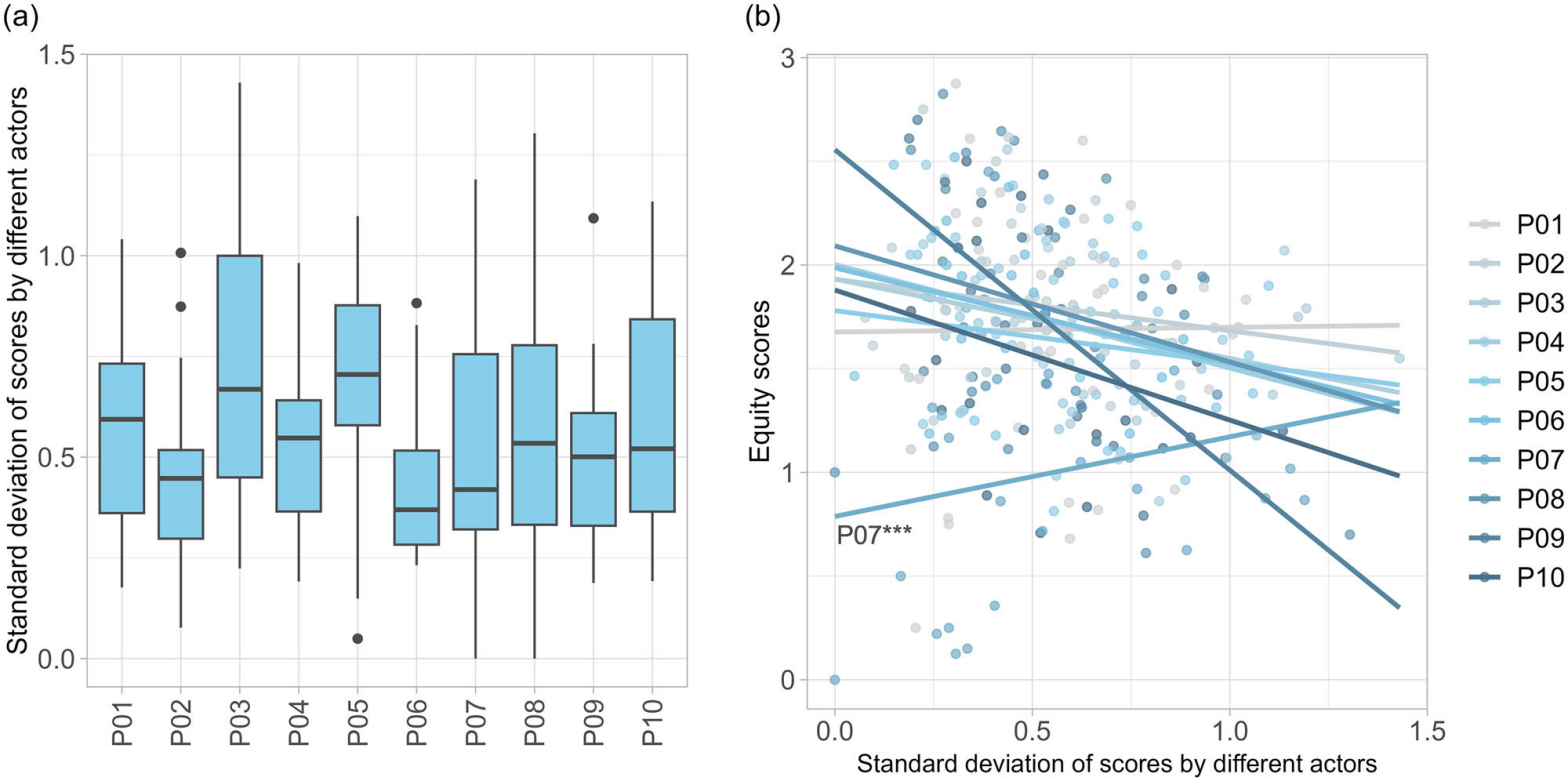
Differences in opinions of actors who participated in SAGE (Site‐level Assessment of Governance and Equity) assessments: (a) distribution of standard deviations between the equity scores by actor groups at different sites by SAGE principle (horizontal lines, median; box, interquartile range [IQR] from Q1 to Q3; whiskers, smallest to largest values within 1.5 × IQR from Q1 and Q3; points, outliers) and (b) correlation between standard deviation and mean equity scores per principle (principles defined in Table 1) (***p < 0.001).

Overall, there was a large variation between different actor groups’ evaluations. With a mean standard deviation of 0.56 across all principles and sites, on average, two thirds of all actors’ responses span a range larger than one equity score (Figure 4a). Participation in decisions (P03) was the principle with the significantly highest disagreement between actor groups on average, with the largest disagreement at a PCA in Lesotho spanning almost the full range of possible scores from 0 to 2.75. Divergence in actor groups’ evaluations was also significantly larger on average for dispute resolution (P05) and lowest for fair law enforcement (P06) and respect for actors (P02) (Appendix S14).

Finally, correlating the mean equity scores with the standard deviation between different actor groups’ evaluations showed that on average more disagreement was associated with lower equity scores (Figure 4b). However, the correlation was statistically positive only for mitigation of negative impacts (P07)—that is, actors seemed to agree more when their evaluations were all rather negative (Appendix S15).

## DISCUSSION

We conducted the first meta‐analysis of SAGE assessments. Overall, the large variability in assessment results between sites is not only clear evidence of the existence of many inequities at the PCAs where SAGE was conducted but also confirms that equity is highly contextual. Such variability mirrors the literature spanning both positive and negative assessments of equity in conservation areas (Bennett et al., 2020; Friedman et al., 2018; Hampton‐Smith et al., 2024; Zafra‐Calvo et al., 2019). Although, as discussed below, the analyses of principles, actor groups, and governance types across the SAGE assessments reveal some important patterns, each site had a unique distribution of equity scores. This confirms that “we need to move beyond over‐simplified and polarized narratives” about whether biodiversity conservation and social well‐being are inherently exclusionary or not (Bennett et al., 2020, p. 9) and illustrates the need for site‐specific governance assessments.

### Most common equity challenges

Collectively, the SAGE assessments identified mitigating negative impacts (P07) of conservation on local communities as the most prevalent equity challenge. There is a striking parallel between the exceptionally negative evaluation of P07 and the empirical emphasis previously found in the conservation literature on the distributional element of equity (Friedman et al., 2018). This conceptual bias has been attributed to the materiality and tangibility of distributive equity and, in direct relation, a methodological overreliance on assessing costs and benefits as more easily measurable (Dawson et al., 2018; Friedman et al., 2018). Yet, the SAGE results show that even when other aspects of procedural and recognitional equity are being considered and assessed, site‐level actors continue to emphasize aspects of distributional equity. Beyond the methodological and conceptual biases in academic debates, negative impacts of conservation seem to remain a primary concern for practitioners and communities.

As at many of the SAGE sites, access or use restrictions and human–wildlife conflict often lie at the core of social–environmental trade‐offs in the protected area model (Coolsaet et al., 2020; Shanko & Tona, 2020; Sharma et al., 2021). In many contexts, negative impacts related to conservation imply substantial and even existential threats to local communities’ livelihoods and survival, whether through human–wildlife conflict, inadequately compensated access restrictions, or uncontrolled extractive threats (Boillat et al., 2010; Brockington & Igoe, 2006; Corbera et al., 2021; Fanari, 2022). As such, among all the SAGE principles, P07 centers the most on fundamental subsistence and often direct trade‐offs, making it one of the most challenging to resolve. Further, the materiality and visibility of negative impacts may contribute to the tendency that site‐level actors were more likely to agree on P07 when the situation was bad.

Although conflict within human–wildlife coexistence may never be completely avoided (Peterson et al., 2010), the way in which such conflicts are negotiated and managed can have a direct impact on social outcomes, people's attitudes, and conservation effectiveness (Hill, 2021). Hence, SAGE describes P07 as mitigation, that is, whether the institutions responsible for mitigating or reducing negative impacts are indeed trying and achieving to do so, instead of the existence or effects of negative impacts per se (IIED, 2023, p. 8). This explicitly frames negative impacts as a question of governance and accountability. Accordingly, many of the explanations given for negative evaluations of P07 in the SAGE reports related to issues of procedure and recognition, including lack of transparency, insufficient political will, poor community participation and awareness over their rights, and lack of consideration of marginalized groups. Although acknowledging their significance, Madden and McQuinn (2014) describe conflicts over negative impacts as the most tangible, visible, and immediate manifestation of disputes as playing out on the surface of (often) deeper level conflicts. In their framework for conservation conflict transformation, they describe 3 levels of conflict, with overt disputes (level 1) often occurring within contexts of preceding unresolved conflicts and histories of resentment and mistrust (level 2) and underpinned by deeply rooted conflicts of identity, disempowerment, and disrespect (level 3). These 3 levels mirror the equity dimensions of distribution, procedure, and recognition. More generally, the literature on human–wildlife conflict has long recognized the importance of centering underlying entanglements of human–human conflicts and so understanding the wider political landscape of how decisions are made and whose priorities are heard and dealt with in response to human–wildlife conflict (Hill, 2021; Peterson et al., 2010). Thus, despite the clear importance of P07, it cannot be addressed without relating it to the other equity principles.

Further, although P07 received such negative evaluations, it was not the only important challenge. Notably, issues of procedure and recognition (i.e., participation in decisions and respect for rights) were also each mentioned as primary challenges by roughly one third of the SAGE reports.

### A case for IP&LC and shared governance

Equity scores tended to be slightly more positive for PCAs governed by and with IP&LC than those governed only by the government, with statistical significance in 4 principles. In line with theoretical expectations, this could be read as evidence in support of IP&LC and shared governance of conservation areas. Indeed, a new IUCN WCPA issues paper understands the governance type as a preexisting enabling condition that influences the possibilities for more equitable processes within PCAs (Franks et al., 2024).

Nonetheless, the differences between the governance types were not only insignificant for all other principles but also smaller in size than one might expect. Besides small sample sizes, we attribute this to the imperfect classification of governance types: sites officially classified as IP&LC or shared governance may in practice remain highly influenced by state or other external authority. The large literature on community‐based conservation has long shown the limited implementation of well‐intended principles, with challenges of top‐down implementation, elite capture, power imbalances in decision‐making, preexisting and ongoing conflicts, and unmet expectations (Abebe et al., 2020; Ahmed & Gasparatos, 2020; Bluwstein et al., 2016; Kawaka et al., 2017). Even in Indigenous territories, external actors, such as NGOs or state agencies, can dominate important decisions over conservation (and beyond) through resource, institutional, and epistemic power (Dawson, Coolsaet, Bhardwaj, Booker, et al., 2024; Reyes‐García et al., 2012, 2014). Accordingly, there is increasing recognition of the variable roles IP&LC representatives can play in area‐based conservation (Dawson, Coolsaet, Bhardwaj, Booker, et al., 2024; Franks et al., 2024; Zhang et al., 2023). Dawson, Coolsaet, Bhardwaj, Booker, et al. (2024) recently developed a typology of IP&LC involvement and authority, ranging from full exclusion, being consultees, stakeholders, or partners, to having primary control or full autonomy. Reviewing a sample of 170 empirical case studies, they were able to show that PCAs with equal partnership or primary control by IP&LC were more likely to report positive ecological outcomes. This is in line with a growing body of scholarship on the contributions of IP&LC stewardship to the effective protection of nature (Adams et al., 2023; Dawson et al., 2021; Dawson, Coolsaet, Bhardwaj, Booker, et al., 2024; Pinto & Dawson, 2023; Reyes‐García et al., 2019, 2022). Although not providing strong evidence in support due to insignificance and small effect sizes, the SAGE results also do not challenge the call for more IP&LC authority in conservation areas.

As important as it is to distinguish the nuanced roles IP&LC play in PCA governance, it is also important to recognize the distinction between IP and LC (IPBES, 2019). In principle, SAGE allows for this level of differentiation between actor groups. Nonetheless, our attempt at separating IP&LC in the analysis to identify potential differences between the 2 showed only marginally significant results. One reason may be that the binary distinction between IP&LC is just as misleading as aggregating them together. Very few SAGE reports explicitly stated whether the participating community groups were Indigenous or not, and, in several sites, SAGE facilitators struggled with assigning the actor groups to one or the other when consulted. For example, in Vietnam, the government uses the term *ethnic minorities* rather than *Indigenous Peoples* (McElwee, 2022) and, specifically at the Vietnamese SAGE site, the local community group included both participants who identified as ethnic minority and others who did not. As recognized at the global policy level, IP&LC definitions are plural, “context‐specific and should be recognized as such” (IPBES, 2019, p. 27). Rather than taking the mostly insignificant results at face value, we attribute them to the conceptually flawed dichotomous distinction between IP&LC and insufficient data from groups where the distinction is clear.

### Importance of different actors’ opinions

The SAGE results showed that PCA management, multistakeholder committees, and IP&LC leaders on average gave higher equity scores across the principles than IP&LC community members, other resource users, and the private sector. Academics also gave lower scores. It may not be surprising that people involved in decision‐making over PCAs are generally more positive in their assessments than people affected by these decisions. However, governance and management assessments in conservation rarely take such different perspectives into account. Although Zafra‐Calvo et al. (2019) did consult a diversity of PCA stakeholders in their first global‐level assessment of equity principles, their respondents included only 16 representatives of IP&LC (6.64%) and 2 private firms (0.8%) among a total sample of 241 (representing an almost even split between government staff, managers, other PA staff, NGOs, and academics). Bennett et al. (2020) in contrast included only local fishers in their assessment of equity in Mediterranean marine protected areas. The extensive representation of both IP&LC and various institutional actors in SAGE confirmed that their perceptions and opinions do differ significantly and uniquely demonstrates the importance of including a diverse range of actors in governance evaluations.

Notably, major differences in perceptions and opinions in this context are often symptomatic of governance challenges, and engaging with them is a first step to addressing them. In our analysis, for most of the principles, lower equity scores tended to come with larger differences in participants’ scoring, though without statistical significance. On the one hand, differences in perception as captured by SAGE demonstrate that people in power either avoid admitting shortcomings or are unaware and insufficiently empathetic of the (severity of) challenges faced by those affected. On the other hand, the people affected by PCAs may have an incentive to provide comparatively negative evaluations as a political stance and to increase chances for future support. Although the SAGE methodology to some degree mitigates dishonest responses as the participants know that they may be required to explain their scores to others in the synthesis workshop, if decision‐making processes and outcomes were perceived as equitable, the incentive for exaggeration should not persist.

The SAGE results showed that PCA users’ evaluations differed not only from those of PCA managers but also from those of multistakeholder committees and IP&LC leaders. This is important given that multistakeholder committees and IP&LC leaders are supposed to represent the interests of PCA users. Yet, elite capture of the positions within these roles and of resources by those in these positions is a well‐known challenge (Bluwstein et al., 2016; Saito‐Jensen et al., 2010; Warren & Visser, 2016). This result highlights the need to consult and listen to voices within communities rather than just community leaders and to recognize that multistakeholder committees, though the main channel for local participation, can end up more aligned with PCA management than community perspectives.

### Limitations and outlook for the future use of SAGE

Our conclusions come with several caveats. First, SAGE, and so this overarching analysis, focuses on equitable governance at the site level. Although larger scale insights can be drawn by aggregating across SAGE sites, the conceptual analysis still relates to decision‐making, relationships, and outcomes at the local level. SAGE does not speak to more fundamental global injustices in the conservation sector, including unequal north–south and core–periphery distributions of costs and benefits, exclusionary decision‐making at the global policy level, postcolonial funding mechanisms, and the epistemic logics of area‐based conservation (Agrawal et al., 2021; Büscher & Fletcher, 2019; Collins et al., 2021; Corson & Campbell, 2023; Domínguez & Luoma, 2020; Kothari, 2021; Massarella et al., 2022). Although some may argue that local‐level inequities cannot be addressed without a transformation of these higher level inequities, evidence of their existence should motivate such higher level transformations.

Second, SAGE simplifies, categorizes, and quantifies political negotiations at conservation sites. Although a wide array of equity issues beyond social impacts are considered, the SAGE questions can capture only specific elements under each principle. Additionally, SAGE does not promote reflection on the underlying causes or contextual, historic, and epistemic power relations at the site level and beyond that maintain identified inequities (Franks & Pinto, 2020). In our meta‐level analysis, further nuance was lost through the high‐level aggregation of assessments. Hence, despite the resulting emphasis on mitigation of negative impacts, the conservation benefits reported from many SAGE sites and the complex and important discussions relating to all other principles at each site should not be dismissed.

Third, our findings are not a representation of the state of equity in conservation around the world. Though large for its kind and spanning a wide range of PCA contexts in geography, ecosystems, and governance types, the sample is still small and not representative. Notably, the sample is skewed with an overrepresentation of sites from Sub‐Saharan African countries, savannah and grassland ecosystems, and government‐governed PCAs. The fact that most negative impacts discussed in the African sites related to human–wildlife conflict and in Latin American sites to uncontrolled external extractive activities is just one example of how conservation governance and conflicts can vary between ecosystems and sociopolitical contexts. As SAGE is scaled up, larger sample sizes in the future should allow for more robust analyses across geographical areas, ecosystem types, and governance arrangements. Similarly, an analysis at the national or regional levels could suggest targeted policy recommendations for a country's PCA system or regional funding bodies.

Finally, because SAGE is conducted only in areas without overt conflict and the sites self‐selected to join this study, our findings relate to comparatively uncontentious PCAs. They do not reflect contexts with deep‐rooted, overt, or violent conflicts. Precisely given this distinction in scope, however, the results are alarming that even within this sample of comparatively inclusive PCAs, important equity challenges remain.

SAGE brings equitable governance to the agenda of conservation actors at the site level and beyond. Though the primary objective needs to remain benefitting local actors, there is potential for SAGE to contribute to reporting against the equitable governance commitment of global conservation targets (CBD, 2022a) and to systematic learning at the national and regional levels. Our analysis is an early‐stage exploration of what can be learned from this still‐small but growing database of SAGE assessments. Our findings should not be taken as a conclusive representation of the state of equity in area‐based conservation, but rather as empirical proof of concept for the importance of considering multiple dimensions and different actors’ viewpoints to meaningfully assess and address social equity at PCAs.

Results of our analyses of 37 SAGE assessments suggest that mitigating the negative impacts of conservation on people remains a primary equity challenge for area‐based conservation at a global scale. This, however, needs to be tackled in relation to the political dynamics of recognition and procedures, especially respect for rights and participation in decision‐making. Further, PCAs governed by or with IP&LC tended to be perceived as slightly more equitable, which supports the case for increased political, institutional, and resource support of IP&LC involvement and autonomy in PCA decision‐making. Yet, for this to be meaningful, barriers to effective community representation and risks of elite capture need to be closely considered, given our finding of common discrepancies between multistakeholder committees and IP&LC leaders and the people they represent. Finally, our meta‐level analysis demonstrated the importance of integrating plural perspectives in governance assessments.

## AUTHOR CONTRIBUTIONS

This study was conducted as part of Naira Dehmel's PhD research supervised by Kate Schreckenberg and Phil Franks. All authors were involved in data collection either as SAGE lead facilitators or coordinators. Data processing, analysis, visualization, and writing were done by Naira Dehmel and substantially reviewed by Kate Schreckenberg, Phil Franks, and Nikoleta Jones. All authors reviewed and edited the final draft.

## Supporting information



Supplementary Materials.
