# Supplementary material for: Insights from equitable governance assessments in conservation areas around the world
Source: Conserv Biol. 2025 Jul 5;40(1):e70101. doi: 10.1111/cobi.70101 (PMC12856824; doi:10.1111/cobi.70101)
Supplement: Supplementary file 1 — Supplementary Materials. [file COBI-40-e70101-s001.docx]

Supporting Information

# Appendix S1 – List of included SAGE sites and excluded data based on misalignment in SAGE questionnaires

| **No** | **Site ID** | **Country** | **Site Name** | **SAGE Version** | **SAGE Report** | **Reasons for excluded or adapted data** |
| --- | --- | --- | --- | --- | --- | --- |
| 1 | BOL_BEN | Reserva de la Biósfera Estación Biológica del Beni | Bolivia | 2 | Yes |  |
| 2 | BOL_MAN | Área Protegida Nacional Manuripi | Bolivia | 2 | Yes |  |
| 3 | BOL_POR | Área Protegida Municipal Área Natural de Manejo Integrado del Bosque de Porvenir | Bolivia | 2 | Yes |  |
| 4 | BOL_PUE | Área Protegida Municipal Bosque Amazónico de Manejo Integral de Puerto Rico | Bolivia | 2 | Yes |  |
| 5 | BOL_TAC | Territorio Indígena TCO Tacana II | Bolivia | 2 | Yes | Scores for questions Q3.2 and Q3.5 by external actors (state institutions and civil society organisations) reversed to reflect that they should have limited influence in an Indigenous Territory. |
| 6 | CMR_BEN | Bénoué National Park | Cameroon | 1 | No |  |
| 7 | CMR_BOU | Bouba Ndjida National Park | Cameroon | 1 | No |  |
| 8 | COL_AND | Parque Municipal Natural Andakí | Colombia | 2 | Yes | Values under principles P05, P07 and P08 excluded as based on less than two questions. |
| 9 | COL_CER | Parque Natural Regional Metropolitano Cerro El Volador | Colombia | 2 | Yes | Values under principles P01, P05, P06 and P10 excluded as based on less than two questions. Values under P07 and P08 excluded as questions were changed substantially. (P07: “Do the communities know the negative impacts their activities can have on the area and do they take any action to mitigate them?” (Without this question only one other question left for P07 that aligns with theme); P08: “Does the community/neighbours know the activities offered to them in the area?”) |
| 10 | GBR_SOL | Solway Firth Marine Protected Area | UK | 1 | Yes | Principles P04 and P07 were excluded for all actor groups as based on less than two questions, and P05 and P08 for “Business” as less than two scores were available for this group. |
| 11 | GBR_STR | Strangford Lough Marine Protected Area | UK | 1 | Yes | Principles P04 and P07 were excluded for all actor groups, and P06 for “Management” and P08 for “Business” and “Other” as less than two scores were available for these groups. |
| 12 | GRC_EAS | National Park of Eastern Macedonia and Thrace | Greece | 1 | Yes | Principles P01 were excluded for “Management” and P09 and P10 for “Locals” as less than two scores were available for these groups. |
| 13 | GRC_EVR | Evros Delta National Park | Greece | 2 | Yes | Principles P01, P05, P06, P09 and P10 were only responded to by facilitator and therefore excluded. |
| 14 | IDN_HUT | Hutan Desa, West Kalimantan | Indonesia | 2 | Yes |  |
| 15 | IRL_CAR | Carlingford Lough Marine Protected Area | Ireland/UK | 2 | Yes | Principles P04 and P07 were excluded as based on less than two questions. |
| 16 | KEN_ELE | Elerai Conservancy | Kenya | 2 | Yes |  |
| 17 | KEN_LUM | Lumo Community Wildlife Conservancy | Kenya | 2 | Yes |  |
| 18 | KEN_SER | Sera Wildlife Conservancy | Kenya | 2 | Yes |  |
| 19 | KHM_KEO | Keo Seima Wildlife Sanctuary | Cambodia | 1 | Yes |  |
| 20 | KHM_VEU | Veun Sai-Siem Pang National Park | Cambodia | 2 | No |  |
| 21 | LSO_SEH | Sehlabathebe National Park | Lesotho | 2 | No |  |
| 22 | MDG_MEN | Northwest coast Menabe (Mangrove du Littoral Nord-Ouest Menabe) | Madagascar | 2 | Yes |  |
| 23 | NGA_OKU | Okumo National Park | Nigeria | 2 | Yes | Principles P01 were excluded for “NGOs and churches”, “Community men” and “Private Sector”, P08 was excluded for “NGOs and churches” and P03 and P08 for “Private Sector” as less than two scores were available for these groups. |
| 24 | PHL_KAL | Mount Kalatungan Range Natural Park | Philippines | 2 | Yes |  |
| 25 | PHL_KIT | Mount Kitanglad Range Natural Park | Philippines | 1 | Yes | Principle P08 was excluded for “NGOs, Academia and researchers” and P10 for “ICCs” as less than two scores were available for these groups. |
| 26 | SYC_POR | Port Glaud District (Port Launay and Baie Ternay MPAs) | Seychelles | 2 | No | Principle P10 was excluded for “Resident /Non MPA users” as less than two scores were available for this group. |
| 27 | TZA_BUR | Burunge Wildlife Management Area | Tanzania | 2 | Yes |  |
| 28 | TZA_MAK | Makame Wildlife Management Area | Tanzania | 2 | Yes |  |
| 29 | TZA_RAN | Randilen Wildlife Management Area | Tanzania | 1 | Yes |  |
| 30 | UGA_KAS | Kasyoha Kitomi Forest Reserve | Uganda | 2 | Yes |  |
| 31 | VNM_DAK | Dak Rong Nature Reserve | Vietnam | 2 | No | Principle P08 was excluded for “Community men” and P04 for “Community women” as less than two scores were available for these groups. |
| 32 | ZMB_LUA | Lower Luano Game Management Area | Zambia | 2 | Yes |  |
| 33 | ZMB_MUF | Mufunta Game Management Area | Zambia | 2 | No |  |
| 34 | ZMB_MUL | Mulobezi Game Management Area | Zambia | 1 | Yes |  |
| 35 | ZMB_NAM | Namwala Game Management Area | Zambia | 2 | Yes |  |
| 36 | ZMB_RUF | Rufunsa Game Management Area | Zambia | 2 | Yes | Principle P05 was excluded for “Community women” and P08 for “Community Resource Board” as less than two scores were available for these groups. |
| 37 | ZWE_MAS | Masoka Conservation Area | Zimbabwe | 2 | No |  |

# Appendix S2 – Clustering of actor groups where not self-explanatory

| **Site ID** | **Name of actor group in SAGE documentation** | **Assigned actor group for analysis** | **Source of additional information** | **Rationale for assignment/ Comment** |
| --- | --- | --- | --- | --- |
| **Names that were not self-explanatory** | | | | |
| BOL_PUE | Authorities | Local Government | SAGE report/profile | This is a municipal area setup by the municipality with support from conservation NGOs, to run under shared governance. This group represents the municipal state institutions who are involved in managing the PA, however, not as the only/main management body. |
| GBR_STR | Other | Academia | SAGE report/profile | SAGE report states that this groups consisted of mainly scientists. |
| GRC_EVR | Central authorities | PCA Management | SAGE report/profile | Based on SAGE report assumed that the central authority includes mainly the "Federal or national ministry or agency" which are listed in the site profile as the governing body. Regional government is then assigned to the Local Government cluster. |
| GRC_EVR | Regional authorities | Local Government | SAGE report/profile | Based on previous comment. |
| GRC_EVR | NP Management Body | Multi-stakeholder Committee | SAGE report/profile | Management Body Board of Directors to which various local stakeholders participate. |
| KEN_LUM | Community Youth | IP youth | SAGE convenor | Community representatives here are officially recognised and self-identify as indigenous |
| KEN_LUM | Community Women | IP women | SAGE convenor | Community representatives here are officially recognised and self-identify as indigenous |
| KEN_LUM | Community Men | IP men | SAGE convenor | Community representatives here are officially recognised and self-identify as indigenous |
| MDG_MEN | Decentralised Territorial Collectivity (CTD) | PCA Management | IIED Coordinator | Apex governance body at site level |
| MDG_MEN | Decentralised Technical Services (STD) | Local Government | IIED Coordinator | These are from government at a local level |
| MDG_MEN | Local community (Vondron'Olona Ifotony, VOI)/COBA | IP Base | SAGE Facilitator | Representatives of local communities who participated in the SAGE assessment are officially recognized as indigenous. These are the Vezo and Sakalava ethnic groups who have inhabited Menabe for hundreds of years. |
| PHL_KAL | Indigenous Cultural Community (ICC) | IP Leaders | IIED Coordinator | Representatives of IP communities that are located within the park |
| PHL_KAL | Barangay Local Government Units/People's Organization | LC Leaders | IIED Coordinator | Local community leaders, although these are actually staff of Local Government as they come from the very lowest level which is very close to the community and could be considered as much if not more grass roots community organisations. Either way they are the way the non-IPs communities are represented in PA governance in the Philippines. |
| PHL_KIT | ICCs | IP Leaders | IIED Coordinator | see ICC at PHL_KAL |
| PHL_KIT | BLGU and other stakeholders | LC Leaders | IIED Coordinator | see BLGU at PHL_KAL |
| SYC_POR | SPGA | PCA Management | Online | Seychelles Parks and Gardens Authority, <https://www.spga.gov.sc/> |
| SYC_POR | Tourism | Private Sector | - | No separate category for tourists, tourists seen as external actors (rather than part of local populations) contributing to the tourist industry, therefore with comparatively aligned interests to the Private Sector tourist industry. |
| SYC_POR | GVI | NGO | Online | Global Vision International (NGO) <https://www.gvi.co.uk/volunteer-in-seychelles/> |
| UGA_KAS | Collaborative Forest Management | Resource users | SAGE Facilitator | These are resource users that access forest resources under resource use agreements. Collaborative Forest Management is a community group that entered into an agreement with the National Forest Authority to sustainably harvest forest resources while offering community support for conservation e.g. participation in the management plans, reporting unauthorized resource use and other decision-making aspects. |
| ZMB_MUL | other stakeholders | NGO | SAGE dataset | Dataset prepared for a previous meta-level analysis of round 1 sites in 2021 that was never published stated "NGO and others" |
| **Groups that included several actors** | | | | |
| BOL_BEN | State actors and Tsimane Indigenous Council | Local Government | IIED Coordinator | Tsimane Indigenous Council is the regional level organisation to represent the Tsimane People. Local Government where in the majority. |
| BOL_BEN | Community leaders and Management Committee | IP Leaders | IIED Coordinator | Most participants were from the Tsimane' Indigenous People, except two leaders from the Movima Indigenous People and one leader from a non-Indigenous peasant community. IP Leaders outnumbered Management Committee members. All Management Committee members were also community leaders. |
| BOL_MAN | Management Committee and Subcentral Chivé | Multi-stakeholder Committee | SAGE report | SAGE report mentions this actor group as 'Management committee' in abbreviation, suggesting that this was the dominant representation. |
| BOL_POR | Authorities and civil society | PCA Management | SAGE report/profile | The ANMI Porvenir, a municipal protected area, has been designated by the Local Government with close support from conservation NGOs. Whilst governance of the area is officially shared, this group represents mainly the driving forces behind the setup and management of the PCA. |
| BOL_PUE | Civil society and academic institutions | NGO | SAGE report/profile | NGOs participated in greater number and played a more important role for the management of the PCA. |
| COL_AND | NGOs, National Natural Parks System, academia | PCA Management | SAGE report/profile | PCA is managed by the Local Government and one NGO (delegated management). Site report states that 1 person from the 'administration of the area', 1 person from the national PA system, and 1 person from the conservation research institute Sinchi participated in this group |
| KHM_KEO | Local authority & DoE | PCA Management | SAGE report/profile | Participants included commune chief, district chief, wildlife sanctuary director, Department of Environment officer and ranger. Dominant actors seem to be part of PCA Management or PCA Management aligned. |
| KHM_KEO | National+NGO+PS | NGO | SAGE report/profile | Site profile lists several NGOs (WCS, CRDT, ELIE, World Hope International) whilst stating the Private Sector to have limited but increasing engagement. Assumption that Private Sector was not so vocal in this group. Ministry of Environment understood as an external conservation actor along with external conservation NGOs. |
| KHM_VEU | NGOs & Private Sectors | NGO | SAGE dataset | Specific NGOs are mentioned several times in the qualitative data, no clear reference to Private Sector. |
| NGA_OKU | PA Management & Federal Govt | PCA Management | SAGE report/profile | PCA Management is led by federal government: The Park is exclusively managed by the Federal government of Nigeria under the supervision of the Federal ministry of Environment through the Department of National Parks Service |
| NGA_OKU | NGOs and churches | NGO | - | No category of churches in meta-level analysis |
| PHL_KAL | Academia and NGO | NGO | SAGE report/profile | 3 participants, site profile suggests a 2 NGO/CBO and 1 academic |
| PHL_KIT | NGOs, Academia and researchers | Academia | SAGE data | Unclear which group was represented more prominently, but qualitative data mentions academia for 3 questions. |
| TZA_RAN | NGO & research | NGO | SAGE dataset | Many mentions of NGOs in the qualitative data, only one mention of academics who have conducted negative impact assessments. |
| ZMB_MUL | PA Management and local govt | PCA Management | SAGE dataset | No indication in dataset which was more prominent in this group, no SAGE report available. PCA Management chosen as the most directly related to the PA and first one listed. |
| ZMB_NAM | NGO and private | NGO | SAGE dataset | In SAGE dataset abbreviated as NGOs, therefore assumed that NGO representatives were in the majority. |
| ZMB_RUF | NGO and private | NGO | SAGE dataset | No indication in report or dataset of whether NGO or Private Sector more prominent in this group, no SAGE report available. NGO chosen as the first one listed. |
| **Excluded actor groups** | | | | |
| BOL_MAN | Environmental Institutions | PCA Management | Facilitator | Excluded as this actor group did not respond to the request for permission to use the data. All other actor groups expressly did want their data to be included in the meta-level analysis. |
| TZA_BUR | New AA council | Multi-stakeholder Committee | IIED Coordinator | The assessment occurred at a time when elections for a new Authorised Association (AA) had just been done so they had both the outgoing AA and incoming AA. The coordinator explained that the incoming had a low opinion of the work of the outgoing one and suggested to only use the outgoing one as the incoming had only been in place a few weeks. |

# Appendix S3 – All equity scores across the sites illustrating the large variation both between principles and actor groups across sites


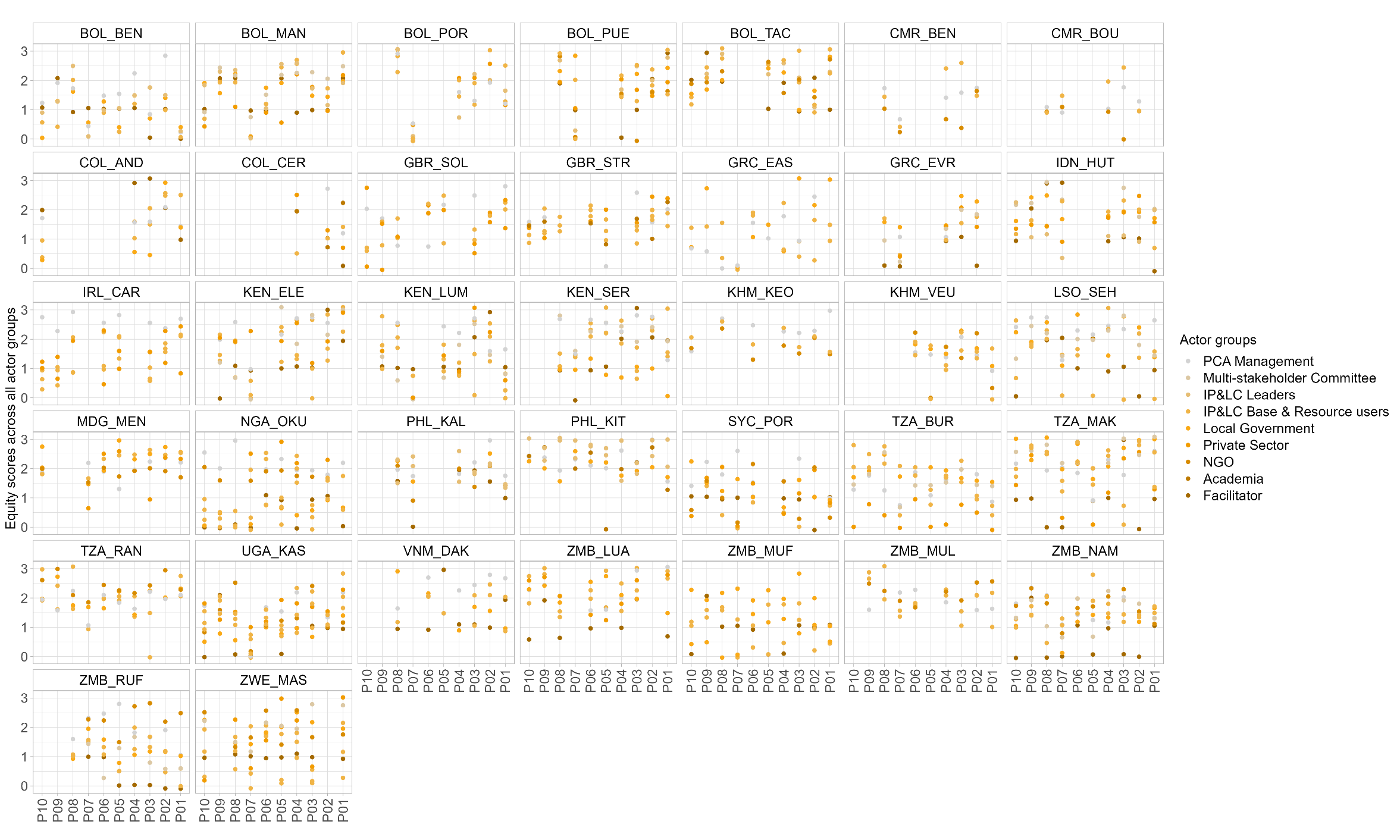


# Appendix S4 - Differences in equity scores by principle

| **Model specification** | Statistical model: | | | | Beta logit regression | | | |
| --- | --- | --- | --- | --- | --- | --- | --- | --- |
|  | Dependent variable: | | | | Normalised mean of equity scores, averaged across all actor groups by principle at each site | | | |
|  | Fixed independent variable: | | | | Equity principles | | | |
|  | Random independent variables: | | | | Country and site (nested) | | | |
|  | Pairwise comparison correction: | | | | Bonferroni | | | |
|  | Postanalysis checks: | | | | Q-Q plot to test normality in residuals | | | |
| **Pairwise comparisons** | Empirical mean | Lower CI (95%) | Upper CI (95%) | Estimated difference to base category (logit scale) | | Standard error (logit scale) | p-value for comparison to base category | Number of observations per category |
| P07 (base) | 1.000 | 0.759 | 1.241 | - | | - | - | 27 |
| P01 | 1.690 | 1.472 | 1.908 | 1.318 | | 0.161 | <.0001 | 34 |
| P02 | 1.823 | 1.673 | 1.973 | 1.541 | | 0.161 | <.0001 | 35 |
| P03 | 1.655 | 1.542 | 1.768 | 1.276 | | 0.160 | <.0001 | 36 |
| P04 | 1.742 | 1.610 | 1.873 | 1.385 | | 0.162 | <.0001 | 34 |
| P05 | 1.608 | 1.424 | 1.792 | 1.233 | | 0.174 | <.0001 | 25 |
| P06 | 1.785 | 1.637 | 1.933 | 1.475 | | 0.176 | <.0001 | 25 |
| P08 | 1.785 | 1.584 | 1.986 | 1.496 | | 0.161 | <.0001 | 33 |
| P09 | 1.787 | 1.561 | 2.013 | 1.493 | | 0.179 | <.0001 | 22 |
| P10 | 1.507 | 1.286 | 1.728 | 1.094 | | 0.176 | <.0001 | 23 |
| *Notes:* Only pairwise comparisons with significant differences are shown. | | | | | | | | |

# Appendix S5 - Differences in equity scores between principles by continent

| **Model specification** | Statistical model: | | | Beta logit regression | | | |
| --- | --- | --- | --- | --- | --- | --- | --- |
|  | Dependent variable: | | | Normalised mean of equity scores, averaged across all actor groups by principle at each site | | | |
|  | Fixed independent variables: | | | Interaction of equity principles with continent | | | |
|  | Random independent variables: | | | Country and site (nested) | | | |
|  | Pairwise comparison correction: | | | Bonferroni | | | |
|  | Postanalysis checks: | | | Q-Q plot to test normality in residuals | | | |
| **Pairwise comparisons** | Empirical mean | Lower CI (95%) | Upper CI (95%) | Estimated difference to base category (logit scale) | Standard error (logit scale) | p-value for comparison to base category | Number of observations per category |
| **Europe:** | | | | | | | |
| P07 (base) | 0.358 | -4.195 | 4.911 | - | - | - | 2 |
| P01 | 2.017 | 1.811 | 2.222 | 5.524 | 0.758 | <.0001 | 4 |
| P02 | 1.755 | 1.679 | 1.831 | 5.114 | 0.751 | <.0001 | 5 |
| P03 | 1.504 | 1.126 | 1.881 | 4.790 | 0.750 | <.0001 | 5 |
| P04 | 1.277 | 0.719 | 1.835 | 4.503 | 0.798 | <.0001 | 2 |
| P05 | 1.509 | 0.881 | 2.138 | 4.85 | 0.754 | <.0001 | 4 |
| P06 | 1.718 | 1.532 | 1.904 | 5.118 | 0.755 | <.0001 | 4 |
| P08 | 1.278 | 0.734 | 1.821 | 4.471 | 0.750 | <.0001 | 5 |
| P09 | 1.309 | 0.994 | 1.625 | 4.585 | 0.756 | <.0001 | 4 |
| P10 | 1.126 | 0.848 | 1.405 | 4.347 | 0.756 | <.0001 | 4 |
|  | | | | | | | |
| P01 (base) | 2.017 | 1.811 | 2.222 | - | - | - | 4 |
| P08 | 1.278 | 0.734 | 1.821 | -1.053 | 0.319 | 0.044 | 5 |
| P10 | 1.126 | 0.848 | 1.405 | -1.177 | 0.336 | 0.02 | 4 |
| **Latin America:** | | | | | | | |
| P07 (base) | 0.410 | -0.239 | 1.058 | - | - | - | 4 |
| P01 | 1.773 | 1.038 | 2.508 | 2.24 | 0.368 | <.0001 | 7 |
| P02 | 1.780 | 1.362 | 2.198 | 2.356 | 0.377 | <.0001 | 7 |
| P03 | 1.637 | 1.339 | 1.936 | 2.053 | 0.378 | <.0001 | 6 |
| P04 | 1.828 | 1.408 | 2.248 | 2.394 | 0.375 | <.0001 | 7 |
| P05 | 1.749 | -0.371 | 3.869 | 2.302 | 0.423 | <.0001 | 3 |
| P06 | 1.244 | 0.529 | 1.958 | 1.863 | 0.478 | 0.004 | 2 |
| P08 | 2.398 | 1.891 | 2.906 | 3.34 | 0.411 | 0.036 | 5 |
| P09 | 1.813 | 0.647 | 2.978 | 2.382 | 0.432 | <.0001 | 3 |
| P10 | 1.067 | 0.470 | 1.663 | 1.345 | 0.412 | 0.05 | 4 |
|  | | | | | | | |
| P08 (base) | 2.398 | 1.891 | 2.906 | - | - | - | 5 |
| P01 | 1.773 | 1.038 | 2.508 | -1.1 | 0.306 | 0.016 | 7 |
| P02 | 1.780 | 1.362 | 2.198 | -0.979 | 0.318 | 0.092 | 7 |
| P03 | 1.637 | 1.339 | 1.936 | -1.282 | 0.319 | 0.003 | 6 |
| P06 | 1.244 | 0.529 | 1.958 | -1.473 | 0.439 | 0.036 | 2 |
| P10 | 1.067 | 0.470 | 1.663 | -1.991 | 0.361 | <.0001 | 4 |
|  | | | | | | | |
| P10 (base) | 1.067 | 0.470 | 1.663 | - | - | - | 4 |
| P02 | 1.780 | 1.362 | 2.198 | 1.012 | 0.312 | 0.053 | 7 |
| P04 | 1.828 | 1.408 | 2.248 | 1.05 | 0.308 | 0.03 | 7 |
| **Sub-Saharan Africa:** | | | | | | | |
| P07 (base) | 1.052 | 0.821 | 1.284 | - | - | - | 18 |
| P01 | 1.623 | 1.269 | 1.977 | 0.85 | 0.160 | <.0001 | 17 |
| P02 | 1.755 | 1.499 | 2.012 | 1.07 | 0.161 | <.0001 | 17 |
| P03 | 1.617 | 1.437 | 1.797 | 0.831 | 0.159 | <.0001 | 19 |
| P04 | 1.735 | 1.563 | 1.908 | 0.985 | 0.159 | <.0001 | 19 |
| P05 | 1.639 | 1.419 | 1.858 | 0.805 | 0.169 | 0.0001 | 15 |
| P06 | 1.791 | 1.598 | 1.984 | 1.014 | 0.170 | <.0001 | 15 |
| P08 | 1.648 | 1.400 | 1.897 | 0.911 | 0.160 | <.0001 | 18 |
| P09 | 1.882 | 1.556 | 2.209 | 1.199 | 0.176 | <.0001 | 13 |
| P10 | 1.658 | 1.356 | 1.960 | 0.861 | 0.179 | 0.0001 | 12 |
| *Notes:* Only pairwise comparisons with significant differences are shown. We attribute insignificant results in the Sout East Asian group to small sample sizes. | | | | | | | |

# Appendix S6 – Number of SAGE reports that mentioned the different principles as a main challenge

# Appendix S7 - Description and ideas for improvement for challenges under P07, P03 and P01

| **Principle** | **Description of the challenges** | **Ideas for improvement** |
| --- | --- | --- |
| **P07** | - External extractive activities and encroachment with negative repercussions on local communities and that are not being impeded by the (PCA) authorities (BOL_BEN, BOL_MAN, BOL_POR, BOL_PUE) - Restricted resource use without effective provision of alternatives (GRC_EVR, IDN_HUT). No strategy for mitigation of negative impacts in place (GRC_EVR, MDG_MEN). - Human-wildlife conflict (KEN_ELE, KEN_LUM, KEN_SER, TZA_MAK, ZMB_MUL, ZMB_NAM, ZMB_RUF): Organisations responsible for compensation/ reparation do not adequately respond (KEN_ELE, KEN_LUM, TZA_MAK, ZMB_MAK). Impacts are being recorded, yet minimal action follows (KEN_ELE). Inadequate skills, human and logistical resources (ZMB_MUL, ZMB_RUF). No compensation (ZMB_NAM), or value of compensation not proportional to the loss (e.g. livestock) (KEN_ELE). Bias in response to certain people (ZMB_MUL, ZMB_RUF). Destruction of crops and houses not recognised for compensation (KEN_ELE). Authorities value wildlife more than humans and their property (KEN_SER). Unawareness among community of compensation process (KEN_SER, ZMB_NAM) | - Develop an impact mitigation strategy (TZA_MAK) - Improve communication and reporting procedures of HWC incidents (ZMB_NAM) - Involvement of local communities in decision-making (IDN_HUT), regular/scheduled assessments with strong community input available to all stakeholders (ZMB_MUL, ZMB_RUF), attendance by authorities in community meetings to report progress of compensations (KEN_SER) - External stakeholders to assist to lobby for the government to respond to destruction of wildlife (KEN_ELE) - Raise awareness on how to claim compensations (KEN_ELE, ZMB_NAM) - Capacity building to upgrade skills on how to act in situations of HWC (ZMB_MUL, ZMB_RUF) - Adequate resourcing for vehicles and field equipment (ZMB_MUL, ZMB_RUF), install electric fences to protect from elephant damages (KEN_ELE), insurance and consolation fees (KEN_LUM), wildlife corridors to minimise contact with community (KEN_SER) - Legal office within the conservancy where HWC can be addressed and devolve resources to lower levels for timely compensation (KEN_SER) - Alternative livelihood projects (MDG_MEN, UGA_KAS) - Avoid preferential treatments (ZMB_MUL, ZMB_RUF) |
| **P03** | - Top-down approach to decision-making without taking stakeholders’ views to account (GBR_STR) - There is no forum for all PCA actors to participate (ZMB_MUL) - Local level elite capture and conflicts over representation and leadership (BOL_POR, BOL_TAC, PHL_KAL) - Limited capabilities and confidence to participate in higher-level decision-making processes (BOL_POR, IDN_HUT) - Limited information sharing for effective participation (BOL_TAC, IDN_HUT, GBR_SOL, GBR_STR, IRL_CAR) - Invitations for only some/ irrelevant decision-making spaces (ICN_HUT), public forums are perceived as merely a formality (GBR_STR) - Marginalised voices are not heard (GBR_STR, KEN_LUM, ZMB_MUL), IP/community issues and concerns hardly raised in comparison to research and project concerns (PHL_KIT) - Limited participation by women and youth, either as excluded due to organisational or cultural reasons (KEN_ELE, KEN_LUM, PHL_KIT, ZMB_MUL), or due to disinterest among youth to participate (BOL_TAC) | - Create a multi-stakeholder forum that is open to all PCA actors (ZMB_MUL) - Devise and implement an inclusive gender strategy (ZMB_MUL) - Interactive training activities and awareness raising for youth (BOL_TAC, KEN_LUM) - Extend invitations and encourage participation (KEN_ELE, KEN_LUM, TZA_MAK) - Meeting minutes to be translated into local language (PHL_KAL) |
| **P01** | - Limited understanding among local communities over their rights (BOL_BEN, IDN_HUT, TZA_BUR) - Lack of information and unclarities over permitted resource use (GBR_STR, IDN_HUT, TZA_BUR) - Differing interpretations of the law (PHL_KIT) - Basic human rights not met (BOL_BEN) - Unclarities in land titles and decision-making at overlapping PAs and Indigenous Territories (BOL_BEN, PHL_KAL, PHL_KIT) - Outsiders do not adhere to free prior informed consent processes (PHL_KAL) - More powerful actors able to disrespect community rights (e.g. investors stop people from grazing, TZA_BUR) - Biased recreational access to the PCA due to unaffordable memberships for less wealthy people (GBR_STR) or insecurity for women, youth and elderly (COL_CER) | - Translation of laws and training events into Indigenous/local languages (BOL_BEN, PHL_KAL, PHL_KIT) - Training and awareness raising about rights, PCA boundaries and permitted resource uses (BOL_BEN, KEN_LUM, PHL_KAL, TZA_BUR, TZA_RAN) - Improve security (more lights, higher police presence, etc.), Create more activities (sport, birding, pedagogical etc) directly tailored at women, youth and elderly (COL_CER) |

# Appendix S8 – Differences in equity scores by governance type and principles

| **Model specification** | Statistical model: | | | | Beta logit regression | | | | | | | |
| --- | --- | --- | --- | --- | --- | --- | --- | --- | --- | --- | --- | --- |
|  | Dependent variable: | | | | Normalised mean of equity scores, averaged across all actor groups by principle at each site | | | | | | | |
|  | Fixed independent variables: | | | | Interaction of equity principles with governance type | | | | | | | |
|  | Random independent variables: | | | | Country and site (nested) | | | | | | | |
|  | Pairwise comparison correction: | | | | Bonferroni | | | | | | | |
|  | Postanalysis checks: | | | | Q-Q plot to test normality in residuals | | | | | | | |
| **Pairwise comparisons** | | Empirical mean | Lower CI (95%) | Upper CI (95%) | | Estimated difference to base category (logit scale) | | Standard error (logit scale) | | p-value for comparison to base category | | Number of observations per category |
| **P05:** | | | | | | | | | | | | |
| Governance by government (base) | | 1.437 | 1.19 | 1.684 | | - | | - | | - | | 12 |
| IP&LC governance | | 2.084 | 1.385 | 2.783 | | 0.865 | | 0.399 | | 0.091 | | 4 |
| **P07:** | | | | | | | | | | | | |
| Governance by government (base) | | 0.871 | 0.418 | 1.324 | | - | | - | | - | | 12 |
| Shared governance | | 1.182 | 0.864 | 1.5 | | 1.217 | | 0.310 | | 0.0003 | | 11 |
| **P08:** | | | | | | | | | | | | |
| Governance by government (base) | | 1.612 | 1.315 | 1.909 | | - | | - | | - | | 17 |
| Shared governance | | 1.977 | 1.59 | 2.364 | | 0.644 | | 0.270 | | 0.051 | | 11 |
| **P09:** | | | | | | | | | | | | |
| Governance by government (base) | | 1.555 | 1.228 | 1.882 | | - | - | | - | | 11 | |
| Shared governance | | 2.141 | 1.676 | 2.606 | | 0.78 | 0.325 | | 0.049 | | 7 | |
| *Notes:* Only pairwise comparisons with significant differences are shown. | | | | | | | | | | | | |

# Appendix S9 – Differences in equity scores by actor group

| **Model specification** | Statistical model: | | | | Beta logit regression | | | | |
| --- | --- | --- | --- | --- | --- | --- | --- | --- | --- |
|  | Dependent variable: | | | | Normalised mean of equity scores by actor group for each principle and site | | | | |
|  | Fixed independent variable: | | | | Actor groups | | | | |
|  | Random independent variables: | | | | Country and site (nested), principles | | | | |
|  | Pairwise comparison correction: | | | | Bonferroni | | | | |
|  | Postanalysis checks: | | | | Q-Q plot to test normality in residuals | | | | |
| **Pairwise comparisons** | | Empirical mean | Lower CI (95%) | Upper CI (95%) | | Estimated difference to base category (logit scale) | Standard error (logit scale) | p-value for comparison to base category | Number of observations per category |
| IP&LC Base and Resource users (base) | | 1.525 | 1.458 | 1.592 | | - | - | - | 541 |
| PCA Management | | 1.874 | 1.792 | 1.956 | | 0.461 | 0.1 | 0.0001 | 241 |
| Multi-stakeholder Committee | | 1.817 | 1.675 | 1.959 | | 0.623 | 0.138 | 0.0002 | 102 |
| IP&LC Leaders | | 1.903 | 1.724 | 2.082 | | 0.603 | 0.167 | 0.008 | 79 |
|  | | | | | | | | | |
| Private Sector (base) | | 1.539 | 1.411 | 1.667 | | - | - | - | 155 |
| PCA Management | | 1.874 | 1.792 | 1.956 | | 0.491 | 0.137 | 0.009 | 241 |
| Multi-stakeholder Committee | | 1.817 | 1.675 | 1.959 | | 0.653 | 0.165 | 0.002 | 102 |
| IP&LC Leaders | | 1.903 | 1.724 | 2.082 | | 0.633 | 0.19 | 0.024 | 79 |
|  | | | | | | | | | |
| Academia (base) | | 1.735 | 1.375 | 2.095 | | - | - | - | 19 |
| PCA Management | | 1.874 | 1.792 | 1.956 | | 1.169 | 0.349 | 0.022 | 241 |
| Multi-stakeholder Committee | | 1.817 | 1.675 | 1.959 | | 1.331 | 0.366 | 0.008 | 102 |
| IP&LC Leaders | | 1.903 | 1.724 | 2.082 | | 1.312 | 0.355 | 0.006 | 79 |
| *Notes:* Only pairwise comparisons with significant differences are shown. | | | | | | | | | |

# Appendix S10 - Differences in equity scores by actor group by continent

| **Model specification** | Statistical model: | | | | Beta logit regression | | | | |
| --- | --- | --- | --- | --- | --- | --- | --- | --- | --- |
|  | Dependent variable: | | | | Normalised mean of equity scores by actor group for each principle and site | | | | |
|  | Fixed independent variable: | | | | Interaction of actor groups with continent | | | | |
|  | Random independent variables: | | | | Country and site (nested), principles | | | | |
|  | Pairwise comparison correction: | | | | Bonferroni | | | | |
|  | Postanalysis checks: | | | | Q-Q plot to test normality in residuals | | | | |
| **Pairwise comparisons** | | Empirical mean | Lower CI (95%) | Upper CI (95%) | | Estimated difference to base category (logit scale) | Standard error (logit scale) | p-value for comparison to base category | Number of observations per category |
| **Europe** | | | | | | | | | |
| Local government (base) | | 1.714 | 1.177 | 2.251 | | - | - | - | 14 |
| PCA Management | | 1.696 | 1.404 | 1.988 | | -1.417 | 0.38 | 0.003 | 36 |
| IP&LC Base & Resource users | | 1.384 | 1.258 | 1.51 | | -1.46 | 0.364 | 0.001 | 84 |
| Private Sector | | 1.473 | 1.251 | 1.695 | | -1.845 | 0.415 | 0.0001 | 40 |
| **Latin America** | | | | | | | | | |
| NGO (base) | | 1.744 | 1.243 | 2.245 | | - | - | - | 14 |
| PCA Management | | 1.594 | 1.301 | 1.887 | | 1.733 | 0.448 | 0.003 | 24 |
| Multi-stakeholder Committee | | 1.925 | 1.479 | 2.371 | | 1.718 | 0.562 | 0.063 | 10 |
| IP&LC Leaders | | 1.694 | 1.423 | 1.965 | | 1.352 | 0.385 | 0.013 | 40 |
| IP&LC Base & Resource users | | 1.739 | 1.529 | 1.949 | | 1.331 | 0.352 | 0.004 | 73 |
| Local government | | 1.56 | 1.305 | 1.815 | | 1.261 | 0.398 | 0.043 | 39 |
| Private Sector | | 1.524 | 1.209 | 1.839 | | 1.231 | 0.422 | 0.0995 | 25 |
| **South-Eastern Asia** | | | | | | | | | |
| IP&LC Base & Resource users (base) | | 1.627 | 1.423 | 1.831 | | - | - | - | 37 |
| Multi-stakeholder Committee | | 2.26 | 1.9 | 2.62 | | 1.802 | 0.536 | 0.022 | 8 |
| IP&LC Leaders | | 2.117 | 1.892 | 2.342 | | 1.4 | 0.358 | 0.003 | 39 |
|  | | | | | | | | | |
| NGO (base) | | 1.59 | 1.294 | 1.886 | | - | - | - | 19 |
| Multi-stakeholder Committee | | 2.26 | 1.9 | 2.62 | | 1.756 | 0.597 | 0.091 | 8 |
| IP&LC Leaders | | 2.117 | 1.892 | 2.342 | | 1.355 | 0.407 | 0.025 | 39 |
|  | | | | | | | | | |
| Academia (base) | | 1.983 | 1.312 | 2.654 | | - | - | - | 9 |
| PCA Management | | 2.109 | 1.938 | 2.28 | | 1.873 | 0.498 | 0.005 | 36 |
| Multi-stakeholder Committee | | 2.26 | 1.9 | 2.62 | | 2.864 | 0.682 | 0.001 | 8 |
| IP&LC Leaders | | 2.117 | 1.892 | 2.342 | | 2.462 | 0.462 | <.0001 | 39 |
| Local Government | | 1.875 | 1.721 | 2.029 | | 1.763 | 0.508 | 0.015 | 36 |
| **Sub-Saharan Africa** | | | | | | | | | |
| IP&LC Base & Resource users  (base) | | 1.504 | 1.417 | 1.591 | | - | - | - | 347 |
| PCA Management | | 1.906 | 1.811 | 2.001 | | 0.632 | 0.128 | <.0001 | 145 |
| Multi-stakeholder Committee | | 1.793 | 1.627 | 1.959 | | 0.65 | 0.155 | 0.0004 | 79 |
| NGO | | 1.703 | 1.555 | 1.851 | | 0.537 | 0.159 | 0.011 | 88 |
|  | | | | | | | | | |
| Private Sector (base) | | 1.567 | 1.37 | 1.764 | | - | - | - | 82 |
| PCA Management | | 1.906 | 1.811 | 2.001 | | 0.544 | 0.184 | 0.048 | 145 |
| Multi-stakeholder Committee | | 1.793 | 1.627 | 1.959 | | 0.562 | 0.203 | 0.086 | 79 |
| *Notes:* Only pairwise comparisons with significant differences are shown. | | | | | | | | | |

# Appendix S11 - Differences in equity scores by actor group and principles

| **Model specification** | Statistical model: | | | | Beta logit regression | | | | | | | |
| --- | --- | --- | --- | --- | --- | --- | --- | --- | --- | --- | --- | --- |
|  | Dependent variable: | | | | Normalised mean of equity scores by actor group for each principle and site | | | | | | | |
|  | Fixed independent variables: | | | | Interaction of actor groups with equity principles | | | | | | | |
|  | Random independent variables: | | | | Country and site (nested) | | | | | | | |
|  | Pairwise comparison correction: | | | | Bonferroni | | | | | | | |
|  | Postanalysis checks: | | | | Q-Q plot to test normality in residuals | | | | | | | |
| **Pairwise comparisons** | | Empirical mean | Lower CI (95%) | Upper CI (95%) | | Estimated difference to base category (logit scale) | | Standard error (logit scale) | | p-value for comparison to base category | | Number of observations per category |
| **P03:** | | | | | | | | | | | | |
| IP&LC Base and Resource users (base) | | 1.33 | 1.128 | 1.532 | | - | | - | | - | | 67 |
| PCA Management | | 2.064 | 1.865 | 2.263 | | 1.489 | | 0.271 | | <.0001 | | 30 |
| Multi-stakeholder Committee | | 2.292 | 1.904 | 2.68 | | 1.918 | | 0.369 | | <.0001 | | 12 |
| Local Government | | 1.755 | 1.458 | 2.052 | | 1.523 | | 0.296 | | <.0001 | | 27 |
| Private Sector | | 1.57 | 1.248 | 1.892 | | 1.446 | | 0.348 | | 0.001 | | 19 |
|  | | | | | | | | | | | | |
| NGOs (base) | | 1.51 | 1.032 | 1.988 | | - | | - | | - | | 16 |
| PCA Management | | 2.064 | 1.865 | 2.263 | | 1.638 | | 0.395 | | 0.001 | | 30 |
| Multi-stakeholder Committee | | 2.292 | 1.904 | 2.68 | | 2.067 | | 0.471 | | 0.0003 | | 12 |
| Local Government | | 1.755 | 1.458 | 2.052 | | 1.672 | | 0.414 | | 0.002 | | 27 |
| Private Sector | | 1.57 | 1.248 | 1.892 | | 1.595 | | 0.453 | | 0.012 | | 19 |
| **P05:** | | | | | | | | | | | | |
| IP&LC Base and Resource users (base) | | 1.487 | 1.238 | 1.736 | | - | | - | | - | | 48 |
| Multi-stakeholder Committee | | 1.8 | 1.275 | 2.325 | | 1.297 | | 0.401 | | 0.034 | | 10 |
| Local Government | | 1.697 | 1.354 | 2.04 | | 0.967 | | 0.320 | | 0.07 | | 20 |
|  | | | | | | | | | | | | |
| Academia (base) | | 0.375 | -4.387 | 5.137 | | - | | - | | - | | 2 |
| PCA Management | | 1.723 | 1.405 | 2.041 | | 3.445 | | 0.837 | | 0.001 | | 21 |
| Multi-stakeholder Committee | | 1.8 | 1.275 | 2.325 | | 4.630 | | 0.871 | | <.0001 | | 10 |
| IP&LC Leaders | | 2.2 | 1.338 | 3.062 | | 3.789 | | 0.971 | | 0.003 | | 5 |
| IP&LC Base and Resource users | | 1.487 | 1.238 | 1.736 | | 3.334 | | 0.804 | | 0.001 | | 48 |
| Local Government | | 1.697 | 1.354 | 2.04 | | 4.3 | | 0.835 | | <.0001 | | 20 |
| Private Sector | | 1.588 | 1.026 | 2.15 | | 3.958 | | 0.899 | | 0.0003 | | 14 |
| NGOs | | 1.63 | 1.056 | 2.204 | | 3.172 | | 0.878 | | 0.008 | | 9 |
| **P07:** | | | | | | | | | | | | |
| IP&LC Base and Resource users (base) | | 0.818 | 0.597 | 1.039 | | - | - | | - | | 47 | |
| PCA Management | | 1.256 | 0.898 | 1.614 | | 1.022 | 0.298 | | 0.017 | | 22 | |
| IP&LC Leaders | | 1.179 | 0.224 | 2.134 | | 1.168 | 0.357 | | 0.03 | | 9 | |
| NGOs | | 1.08 | 0.637 | 1.523 | | 1.732 | 0.397 | | 0.0004 | | 13 | |
|  | | | | | | | | | | | | |
| Private Sector (base) | | 0.71 | 0.254 | 1.166 | | - | - | | - | | 14 | |
| NGOs | | 1.08 | 0.637 | 1.523 | | 1.405 | 0.466 | | 0.073 | | 13 | |
| **P08:** | | | | | | | | | | | | |
| IP&LC Leaders (base) | | 2.461 | 1.993 | 2.929 | | - | - | | - | | 10 | |
| IP&LC Base and Resource users | | 1.652 | 1.452 | 1.852 | | -1.198 | 0.381 | | 0.035 | | 61 | |
| Local Government | | 1.745 | 1.385 | 2.105 | | -1.508 | 0.418 | | 0.007 | | 23 | |
| NGOs | | 1.858 | 1.444 | 2.272 | | -1.471 | 0.508 | | 0.08 | | 12 | |
| **P09:** | | | | | | | | | | | | |
| Private Sector (base) | | 1.475 | 1.059 | 1.891 | | - | - | | - | | 14 | |
| NGOs | | 2.044 | 1.496 | 2.592 | | 1.714 | 0.544 | | 0.045 | | 7 | |
| **P10:** | | | | | | | | | | | | |
| Private Sector (base) | | 1.182 | 0.671 | 1.693 | | - | - | | - | | 14 | |
| PCA Management | | 1.853 | 1.59 | 2.116 | | 1.32 | 0.437 | | 0.071 | | 18 | |
| IP&LC Leaders | | 1.9 | 0.954 | 2.846 | | 1.968 | 0.593 | | 0.025 | | 5 | |
| IP&LC Base and Resource users | | 1.295 | 1.069 | 1.521 | | 1.187 | 0.365 | | 0.032 | | 41 | |
| *Notes:* Only pairwise comparisons with significant differences are shown. | | | | | | | | | | | | |

# Appendix S12 – Differences in equity scores by IP&LC youth, men and women per principle

| **Model specification** | Statistical model: | | | | Beta logit regression | | | | | | | |
| --- | --- | --- | --- | --- | --- | --- | --- | --- | --- | --- | --- | --- |
|  | Dependent variable: | | | | Normalised mean of equity scores by IP&LC youth, men and women for each principle and site | | | | | | | |
|  | Fixed independent variables: | | | | Interaction of actor groups with equity principles | | | | | | | |
|  | Random independent variables: | | | | Country and site (nested) | | | | | | | |
|  | Pairwise comparison correction: | | | | Bonferroni | | | | | | | |
|  | Postanalysis checks: | | | | Q-Q plot to test normality in residuals | | | | | | | |
| **Pairwise comparisons** | | Empirical mean | Lower CI (95%) | Upper CI (95%) | | Estimated difference to base category (logit scale) | | Standard error (logit scale) | | p-value for comparison to base category | | Number of observations per category |
| **P01:** | | | | | | | | | | | | |
| IP&LC youth (base) | | 1.55 | -0.183 | 3.283 | | - | | - | | - | | 5 |
| IP&LC men | | 1.363 | 0.901 | 1.825 | | - 1.106 | | 0.504 | | 0.085 | | 20 |
| **P03:** | | | | | | | | | | | | |
| IP&LC youth (base) | | 0.35 | -0.169 | 0.869 | | - | | - | | - | | 5 |
| IP&LC men | | 1.494 | 1.108 | 1.88 | | 1.556 | | 0.581 | | 0.022 | | 21 |
| **P05:** | | | | | | | | | | | | |
| IP&LC youth (base) | | 1.4 | 0.09 | 2.71 | | - | | - | | - | | 5 |
| IP&LC women | | 1.698 | 1.297 | 2.099 | | -1.435 | | 0.613 | | 0.058 | | 14 |
| IP&LC men | | 1.279 | 0.764 | 1.794 | | -2.05 | | 0.577 | | 0.001 | | 17 |
| **P08:** | | | | | | | | | | | | |
| IP&LC youth (base) | | 1.05 | 0.041 | 2.059 | | - | - | | - | | 5 | |
| IP&LC women | | 1.836 | 1.473 | 2.199 | | 1.5 | 0.605 | | 0.039 | | 18 | |
| IP&LC men | | 1.862 | 1.423 | 2.301 | | 1.287 | 0.582 | | 0.081 | | 19 | |
| *Notes:* Only pairwise comparisons with significant differences are shown. | | | | | | | | | | | | |

# Appendix S13 - Differences in equity scores by IP vs LC Leaders and Base per principle

| **Model specification** | Statistical model: | | | | Beta logit regression | | | | | | | |
| --- | --- | --- | --- | --- | --- | --- | --- | --- | --- | --- | --- | --- |
|  | Dependent variable: | | | | Normalised mean of equity scores by IP and LC (Leaders and Base) for each principle and site | | | | | | | |
|  | Fixed independent variables: | | | | Interaction of actor groups with equity principles | | | | | | | |
|  | Random independent variables: | | | | Country and site (nested) | | | | | | | |
|  | Pairwise comparison correction: | | | | Bonferroni | | | | | | | |
|  | Postanalysis checks: | | | | Q-Q plot to test normality in residuals | | | | | | | |
| **Pairwise comparisons** | | Empirical mean | Lower CI (95%) | Upper CI (95%) | | Estimated difference to base category (logit scale) | | Standard error (logit scale) | | p-value for comparison to base category | | Number of observations per category |
| **P01:** | | | | | | | | | | | | |
| IP Leaders and Base (base) | | 1.527 | 0.771 | 2.283 | | - | | - | | - | | 12 |
| LC Leaders and Base | | 1.672 | 1.439 | 1.905 | | 0.703 | | 0.405 | | 0.083 | | 55 |
| **P03:** | | | | | | | | | | | | |
| IP Leaders and Base (base) | | 0.752 | 1.387 | 2.343 | | - | | - | | - | | 12 |
| LC Leaders and Base | | 0.836 | 1.12 | 1.564 | | -0.94 | | 0.458 | | 0.04 | | 57 |
| **P08:** | | | | | | | | | | | | |
| IP Leaders and Base (base) | | 2.462 | 2.114 | 2.81 | | - | | - | | - | | 10 |
| LC Leaders and Base | | 1.705 | 1.479 | 1.931 | | -0.825 | | 0.488 | | 0.091 | | 53 |
| **P10:** | | | | | | | | | | | | |
| IP Leaders and Base (base) | | 1.141 | 0.616 | 1.666 | | - | - | | - | | 7 | |
| LC Leaders and Base | | 1.495 | 1.209 | 1.781 | | 1.086 | 0.632 | | 0.086 | | 32 | |
| *Notes:* Only pairwise comparisons with significant differences are shown. | | | | | | | | | | | | |

# Appendix S14 - Standard deviation between actor groups’ scores by principle

| **Model specification** | Statistical model: | | | | Linear mixed regression | | | |
| --- | --- | --- | --- | --- | --- | --- | --- | --- |
|  | Dependent variable: | | | | Standard deviation between actor groups’ scores by principle at each site | | | |
|  | Fixed independent variable: | | | | Equity principles | | | |
|  | Random independent variables: | | | | Country and site (nested) | | | |
|  | Pairwise comparison correction: | | | | Bonferroni | | | |
|  | Postanalysis checks: | | | | Q-Q plot to test normality in residuals | | | |
| **Pairwise comparisons** | Empirical mean | Lower CI (95%) | Upper CI (95%) | Estimated difference to base category | | Standard error | p-value for comparison to base category | Number of observations per category |
| P03 (base) | 0.731 | 0.643 | 0.819 | - | | - | - | 36 |
| P02 | 0.443 | 0.354 | 0.532 | -0.289 | | 0.054 | <.0001 | 35 |
| P04 | 0.527 | 0.437 | 0.617 | -0.205 | | 0.054 | 0.009 | 34 |
| P06 | 0.433 | 0.331 | 0.535 | -0.299 | | 0.059 | <.0001 | 25 |
| P07 | 0.551 | 0.452 | 0.650 | -0.181 | | 0.058 | 0.09 | 27 |
| P08 | 0.553 | 0.462 | 0.644 | -0.178 | | 0.055 | 0.055 | 33 |
| P09 | 0.492 | 0.385 | 0.600 | -0.239 | | 0.062 | 0.006 | 22 |
| P05 (base) | 0.677 | 0.575 | 0.779 | - | | - | - | 25 |
| P02 | 0.443 | 0.354 | 0.532 | -0.234 | | 0.06 | 0.005 | 35 |
| P06 | 0.433 | 0.331 | 0.535 | -0.244 | | 0.064 | 0.008 | 25 |
| *Notes:* Only pairwise comparisons with significant differences are shown. | | | | | | | | |

# Appendix S15- Correlation between mean equity scores and standard deviation between actor groups' score by principle

| **Model specification** | Statistical model: | | Beta logit regression | | |
| --- | --- | --- | --- | --- | --- |
|  | Dependent variable: | | Normalised mean of equity scores, averaged across all actor groups by principle at each site | | |
|  | Fixed independent variable: | | Interaction between standard deviation between actor groups’ scores by principle and equity principles | | |
|  | Random independent variables: | | Country and site (nested) | | |
|  | Number of observations | | 294 | | |
|  | Postanalysis checks: | | Q-Q plot to test normality in residuals | | |
| **Pairwise comparisons** | Correlation coefficient estimate | Standard error of coefficient | | z-value | p-value |
| Standard Deviation | -0.074 | 0.424 | | -0.173 | 0.8623 |
| P07 (to base of P01) | -2.377 | 0.348 | | -6.839 | <.0001 |
| Standard Deviation * P07 (to base of P01) | 2.024 | 0.553 | | 3.657 | 0.0003 |
| *Notes:* Only pairwise comparisons with significant differences are shown. | | | | | |
